# Systematic comparison of household income, consumption, and assets to measure health inequalities in low- and middle-income countries

**DOI:** 10.1038/s41598-024-54170-1

**Published:** 2024-02-15

**Authors:** Mathieu J. P. Poirier

**Affiliations:** 1 Global Strategy Lab, Dahdaleh Institute for Global Health Research, Faculty of Health, York University, 4700 Keele Street, Dahdaleh Building 2120, Toronto, ON M3J 1P3 Canada; 2 School of Global Health, Faculty of Health, York University, Toronto, Canada

**Keywords:** Malnutrition, Epidemiology, Risk factors, Health care economics, Public health, Epidemiology, Health policy

## Abstract

There has been no systematic comparison of how the three most common measures to quantify household SES—income, consumption, and asset indices—could impact the magnitude of health inequalities. Microdata from 22 Living Standards Measurement Study surveys were compiled and concentration indices, relative indices of inequality, and slope indices of inequality were calculated for underweight, stunting, and child deaths using income, consumption, asset indices, and hybrid predicted income. Meta-analyses of survey year subgroups (pre-1995, 1995–2004, and post-2004), outcomes (child deaths, stunting, and underweight), and World Bank country-income status (low, low-middle, and upper-middle) were then conducted. Asset indices and the related hybrid income proxy result in the largest magnitudes of health inequalities for all 12 overall outcomes, as well as most country-income and survey year subgroupings. There is no clear trend of health inequality magnitudes changing over time, but magnitudes of health inequality may increase as country-income levels increase. There is no significant difference between relative and absolute inequality measures, but the hybrid predicted income measure behaves more similarly to asset indices than the household income it is supposed to model. Health inequality magnitudes may be affected by the choice of household SES measure and should be studied in further detail.

## Introduction

All bivariate measures of health inequality require two variables—a health outcome and a measure of socioeconomic status (SES). In the field of global health, much scrutiny has been paid to the way health outcomes are measured, modeled, scaled, weighted, and quantified; but relatively little research has been conducted on how different methods of measuring SES itself can impact magnitudes of health inequalities. Despite the myriad of methods that have been employed to measure SES in global health, there has not been a systematic comparison how these choices impact magnitudes of social health inequality. In order to address this gap in the global health literature, this study empirically evaluates how four different measures of SES affect the magnitude of wealth-related health inequalities across 22 nationally representative household surveys conducted in low and middle-income countries (LMICs).

The three most widely used measures of SES that are used to calculate health inequalities in global health are income, consumption, and asset indices^1^. Income is the primary method of quantifying SES in high-income countries, but in many LMICs, income can be highly variable from month to month, may be incorrectly reported by survey respondents, and may be an inaccurate signifier of a household’s SES if a large part of a household’s spending comes from savings or loans^2–5^. One widely utilized solution to many of these issues in international household surveys and development literature is to measure households’ expenditures over a certain time interval, often broken down into broad consumption categories. Proponents of these household consumption measures cite advantages of capturing the impacts of income smoothing through savings and loans, resulting in measures that are more consistent from month to month, and that may be more representative of a household’s permanent SES^6,7^. In practice, however, household expenditure data usually takes at least an hour to collect, resulting in lengthy and expensive surveys, and even then, may be affected by recall bias, observer bias, and high attrition rates^5,8^.

In response to these challenges, a method of quantifying a household’s assets into a single SES index was developed using household assets widely available in standardized household surveys in a seminal work by Filmer and Pritchett^9^. Asset indices are now the most widely used method to quantify SES in global health household surveys of LMICs because assets can be quickly and objectively measured by surveyors, remain relatively stable over time, and pre-calculated indices are now included in the most widely used health surveys including Demographic and Health Surveys (DHS) and Multiple Indicator Cluster Surveys (MICS)^5,10,11^. Despite their relative speed and ease of collection, there has been considerable debate over how best to calculate asset indices^12–15^ and the resulting measures can be difficult to interpret due to the lack a meaningful interval scale^9,16^. Because they do not have an interval scale, asset indices can only be used to compare relative orderings of households across contexts and over time, and only if care is taken to account for changes in the social value of household assets such as smartphones or access to sanitation^17,18^.

A more recent innovation to address asset indices’ lack of scale proposes to simulate household income by assigning each centile of the SES spectrum—as ranked by asset indices—a country- and year-specific simulated income distribution^19^. The researchers developing this method justify its use with the argument that relative rankings of households according to asset wealth and income are generally similar, and the simple step of interpolating income distribution data allows us to convert asset indices to a meaningful context-specific interval scale. Although this method has shown promise when applied to household surveys in LMICs^20^, there is no published systematic comparison with real-world household income and consumption-based health inequality measures. This means that although relative household rankings are based on the asset index and absolute differences between those households are based on simulated income distributions, there is little evidence of whether this new construct results in health inequality magnitudes that more closely resemble those based on asset indices or on household income.

Although other measures of SES such as education, social class, subjective social standing, and multidimensional poverty have been used to measure health inequalities; income, consumption, and asset indices are unique in that they share a common goal of measuring social standing through financial well-being regardless of country or social institutions present in those countries. Education level is widely used as a proxy of SES, especially in lower income countries, but takes aim at a different dimension of social wellbeing than financial indicators. Educational strata can be affected by methodological issues such as assumptions that each year of education is equally indicative of an increase in SES and is of equal quality for each student^5^. For the purposes of proxying household SES, what is even more important than the incomparability of education levels across even sub-national jurisdictions is the fact that it is often more indicative of community-level social development than of household-level SES.

No matter which of these measures is used, each has a legitimate claim to measuring at least one real dimension of household SES while also suffering from some degree of theoretical and practical disadvantage. Nevertheless, many authors have taken the explicit or implicit assumption that one method is superior to other alternatives rather than empirically studying the effect each method has on the magnitude of social health inequalities^1,20^. This study makes no normative assumption that any method of measuring SES is implicitly superior, nor that measures that result in larger magnitudes of wealth-related inequalities in health are more accurately representing household SES. Rather, each measure has utility for global health research that is contingent on careful measurement and interpretation.

Despite the possibility that SES measures can have a large impact on the magnitude of social health inequalities, a critical interpretive synthesis of existing literature^21^ found that only three studies have compared the use of different methods with the same microdata in more than one country, and none have compared all three measures of income, consumption, and asset indices. The largest study of its type conducted by Wagstaff and Watanabe^22^ compared equivalized household consumption with asset indices using Living Standards Measurement Study (LSMS) data in 19 countries. Their findings suggest that there was likely no difference between the two measures, although significant differences were found in fewer than a quarter of the cases, with concentration indices of underweight and stunting found to be slightly larger using consumption. Another study by Sahn and Stifel^1^ predicted standardized anthropometric height-for-age Z-scores for 12 country-years, finding little difference between the two measures, but highlighting cases where the asset index did point to larger inter-quintile (rich-poor) differences than consumption. Filmer and Scott^23^ analyzed the ratio of child deaths to births, finding that per capita expenditures result in smaller inter-quintile differences than asset indices in four out of eight countries analyzed, with the remainder having no significant difference. Although not a primary study of SES measures’ impacts on health inequalities, Howe et al^24^. review of asset index and consumption concordance (including single-country studies) found that health inequalities were larger for asset indices in three studies, larger for consumption in two studies, with one study finding mixed results. In sum, the few studies that have compared wealth-related health inequalities using both consumption and asset indices have resulted in conflicting conclusions.

Howe et al^24^. speculate on reasons for this discordance. Among the entire 17 study set included for analysis, there was higher agreement between consumption and asset indices in middle income settings, urban areas, and when more and diverse indicators were included in asset indices. Country-income level could therefore theoretically affect asset index performance if a household’s spending on non-asset goods, such as food expenses, is systematically correlated with country-level income^23^. Alternatively, if the amount of household spending that is captured by asset indices increases as countries become richer, then asset index comparability with income and consumption will tend to increase as time goes on due to the general tendency of country income-levels to increase. Relatedly, the tendency of asset prices to fall as importation of cheaper household durables from emerging markets has become more common may result in a divergence between asset wealth and both income and consumption^25^. Bivariate health inequality measures also depend heavily on the health outcome being measured. There is evidence, for example, that inequality in child mortality measured using the DHS asset index becomes larger with increasing urbanization^26^ or when countries decrease their overall rates of child mortality over time^27^. This means that the effect of global improvements in child mortality and other health outcomes^28^ may systematically affect the measurement of wealth-related health inequalities over time.

Lastly, the methods used to calculate social health inequalities influence the conclusions that are reached, and especially depending on whether relative or absolute differences are emphasized. In theory, measures of absolute difference, such as interquartile differences or slope index of inequality (SII), would result in greater inequalities and be more sensitive to differences in scale of measures of SES^29^. The concentration index, which measures relative inequality and may be more sensitive to changes in health outcomes at the middle of the SES spectrum can be affected by different orderings of households depending on the measure of SES used^30,31^. If the goal of these summary measures is to capture the entirety of the SES spectrum, then some measures such as the concentration index, the relative index of inequality (RII), and the SII are more appropriate than other measures such as interquartile differences, but generally, each measure can be said to represent different normative judgements applied to the measurement of health inequalities^32,33^. In sum, the methods by which inequality is calculated, the health outcomes under study, the year in which the study is conducted, and the country-income level of the population may all affect how different SES measures affect inequalities in health.

To establish a baseline association among the three primary measures of SES, this study compiled 22 country-years of LSMS data and calculated concentration indices, RIIs, and SIIs for child deaths, stunting, and underweight using household income, consumption, and asset indices as measures of SES. Every publicly available survey containing data on income, consumption, household assets, and child health outcomes was then systematically compiled, followed by calculating asset indices and assigning a hybrid income proxy according to the relevant country-year. Three measures of social health inequality—the concentration index, RII, and SII—were then calculated for each outcome, after which the magnitudes of each summary measure were compared using meta-analytic techniques and broken down according to survey year, country-income level, and health outcome to investigate the ways in which each SES measure may be shifting across time and space.

## Results

**Figure 1:**
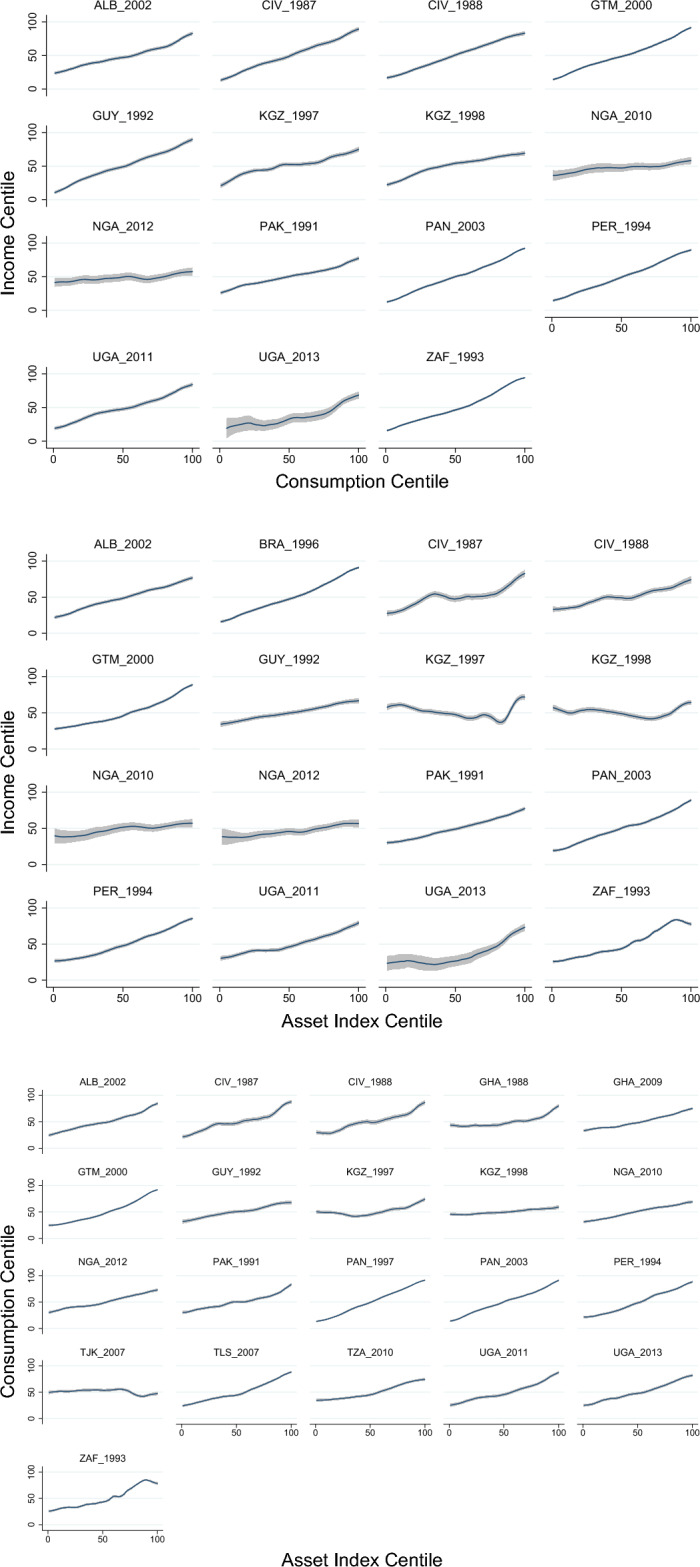
Kernel-weighted local polynomial plot of income centiles vs consumption centiles (top), income centiles vs asset index centiles (middle), and consumption centiles vs asset index centiles (bottom). No graphs of the predicted income hybrid measure were generated because these would have been identical to asset index graphs.

In most countries, there was a positive association between all three measures of income, consumption, and the asset index. Centiles of income, consumption, and asset indices were plotted against alternate measures for each country in Fig. 1 to examine the strength of association at different points in the SES distribution. There is a fairly strong and monotonic increase in income for an equivalent increase in consumption in almost every country, with the exception of a relatively flatter distribution in Nigeria for both 2010 and 2012; possibly due to the relatively small sample sizes for Nigerian income data (N = 501; 471, respectively). Consumption centiles are also positively associated with asset centiles, albeit somewhat less monotonically, except for a small decrease at the top of the South African asset wealth spectrum and a flat distribution with small decrease at the top of the Tajikistani asset wealth spectrum. Finally, income centiles display a highly variable association with asset index centiles in Cote d’Ivoire and Kyrgyzstan, although a positive overall association is maintained. There is no inherent reason to expect strong concordance between these measures in every context, but the strength of agreement between all three measures is notable and indicates that all three measures of SES are related at some level.

**Figure 2:**
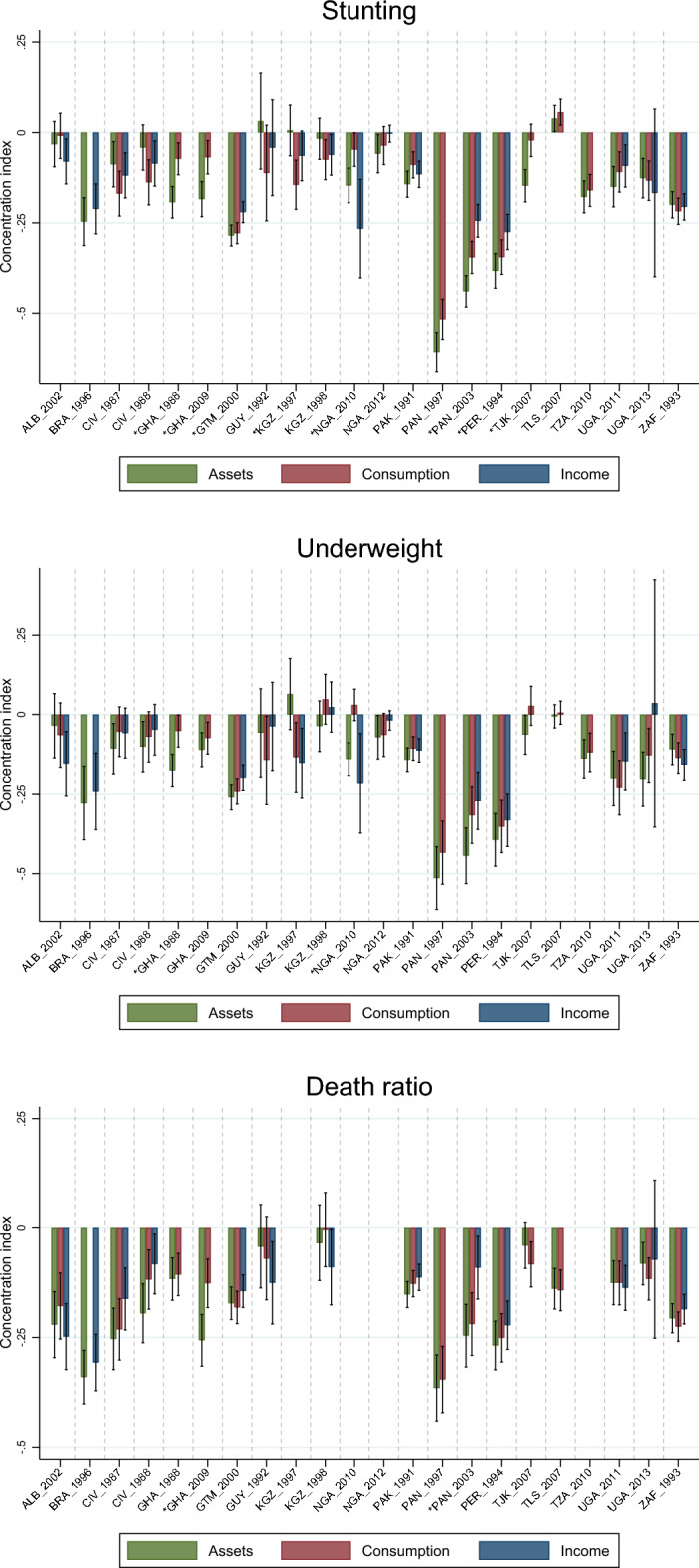
Concentration indices for stunting (top), underweight (middle), and death ratio (bottom) for every country-year and SES measure with 95% confidence intervals.

Disaggregated health outcome prevalence for stunting, underweight, and child death ratios are presented for survey-specific quintiles in Supplementary Information Tables S1-S2. As expected, there is a decline in prevalence for nearly every outcome as quintiles increase using all three SES measures. A simple proxy of national poverty lines based on the lowest quintiles of households ordered by assets, consumption, and income reveals differences in the concentration of each outcome (Supplementary Information Fig. S1). A household asset-based poverty line most frequently results in the highest prevalence of underweight (12 of 22 country-years), while a consumption-based poverty line results in the highest prevalence of stunting (11 of 22 country-years) and mean death ratio (10 of 18 country-years). Moving beyond this simple poverty-based approach, survey-specific social inequality measures for stunting, underweight, and child death ratios are presented for income, consumption, and asset indices using concentration indices in Fig. 2, and in greater detail for all inequality measures in Supplementary Information Tables S3-S11 and Fig. S2.

Concentration index values are larger in magnitude (i.e., higher inequality) using asset indices for all three outcomes. Overall, there are significant concentration index differences between SES measures in 12 of 52 comparisons for stunting, 2 of 52 comparisons for underweight, and 2 of 42 comparisons for child deaths. RII values are larger in magnitude (i.e., higher inequality) using hybrid income proxies for all three outcomes. Overall, there are significant RII differences between SES measures in 9 of 52 comparisons for stunting, 4 of 52 comparisons for underweight, and 0 of 42 comparisons for child deaths. SII values are larger in magnitude (i.e., higher inequality) using asset indices for all three outcomes. Overall, there are significant SII differences between SES measures in 11 of 51 comparisons for stunting, 4 of 49 comparisons for underweight, and 0 of 42 comparisons for child deaths. Most inequality indices were not significantly different from each other, but when they were, asset index-derived indices most often resulted in larger inequalities.

Meta-analysis of all concentration indices indicates no significant difference in the magnitude of inequality between SES measures for outcomes of stunting, underweight, and child deaths (Table 1; Supplementary Information Fig. S3). Combining all health outcomes for meta-analysis does not change the result, but asset indices result in the largest magnitudes of inequality for every outcome. Meta-analysis of RIIs (Table 2; Supplementary Information Fig. S4) reveals a very similar pattern, with all SES measures resulting in magnitudes of inequality that are not statistically different. The hybrid income proxy, which is based on the household asset index, results in the largest magnitudes of inequality for all RII outcomes. Finally, SII results also indicate non-statistically different magnitudes of inequality for every outcome and SES measure (Table 3; Supplementary Information Fig. S5). Like the RII, the SII is largest in magnitude using the hybrid income proxy for all four outcomes.

**Table 1:** Meta-analysis of aggregated concentration index results.

	Death ratio			Stunting			Underweight			Combined		
	CI	LCI	UCI	CI	LCI	UCI	CI	LCI	UCI	CI	LCI	UCI
Assets Consumption Income	− 0.18 − 0.16 − 0.16	− 0.22 − 0.19 − 0.19	− 0.14 − 0.13 − 0.12	− 0.17 − 0.15 − 0.14	− 0.23 − 0.20 − 0.19	− 0.10 − 0.09 − 0.10	− 0.16 − 0.12 − 0.14	− 0.21 − 0.17 − 0.19	− 0.11 − 0.07 − 0.10	− 0.17 − 0.14 − 0.15	− 0.20 − 0.17 − 0.17	− 0.14 − 0.11 − 0.13
L Assets L Consumption L Income LM Assets LM Consumption LM Income UM Assets UM Consumption UM Income	− 0.11 − 0.11 − 0.12 − 0.23 − 0.20 − 0.17 − 0.26 − 0.22 − 0.20	− 0.16 − 0.13 − 0.14 − 0.28 − 0.25 − 0.22 − 0.35 − 0.25 − 0.30	− 0.06 − 0.09 − 0.09 − 0.17 − 0.16 − 0.12 − 0.18 − 0.19 − 0.09	− 0.12 − 0.10 − 0.09 − 0.18 − 0.17 − 0.15 − 0.30 − 0.28 − 0.22	− 0.16 − 0.12 − 0.12 − 0.31 − 0.29 − 0.22 − 0.46 − 0.41 − 0.25	− 0.08 − 0.07 − 0.07 − 0.05 − 0.04 − 0.08 − 0.14 − 0.16 − 0.19	− 0.12 − 0.09 − 0.08 − 0.18 − 0.14 − 0.16 − 0.28 − 0.22 − 0.21	− 0.16 − 0.13 − 0.15 − 0.28 − 0.24 − 0.24 − 0.50 − 0.40 − 0.29	− 0.08 − 0.04 − 0.02 − 0.08 − 0.04 − 0.07 − 0.06 − 0.05 − 0.14	− 0.12 − 0.09 − 0.10 − 0.19 − 0.17 − 0.16 − 0.28 − 0.24 − 0.21	− 0.14 − 0.11 − 0.12 − 0.26 − 0.23 − 0.20 − 0.35 − 0.30 − 0.25	− 0.09 − 0.08 − 0.08 − 0.13 − 0.11 − 0.12 − 0.20 − 0.18 − 0.17
Pre-1995 Assets Pre-1995 Consumption Pre-1995 Income 1995–2004 Assets 1995–2004 Consumption 1995–2004 Income Post-2005 Assets Post-2005 Consumption Post-2005 Income	− 0.18 − 0.16 − 0.15 − 0.23 − 0.19 − 0.18 − 0.13 − 0.12 − 0.13	− 0.23 − 0.22 − 0.19 − 0.32 − 0.28 − 0.26 − 0.19 − 0.14 − 0.18	− 0.14 − 0.11 − 0.11 − 0.15 − 0.10 − 0.10 − 0.06 − 0.10 − 0.08	− 0.15 − 0.17 − 0.15 − 0.23 − 0.23 − 0.15 − 0.12 − 0.06 − 0.12	− 0.23 − 0.24 − 0.21 − 0.39 − 0.36 − 0.22 − 0.18 − 0.12 − 0.24	− 0.07 − 0.09 − 0.09 − 0.08 − 0.10 − 0.08 − 0.06 − 0.01 0.00	− 0.16 − 0.13 − 0.13 − 0.22 − 0.19 − 0.16 − 0.11 − 0.06 − 0.15	− 0.22 − 0.19 − 0.20 − 0.35 − 0.32 − 0.25 − 0.16 − 0.12 − 0.22	− 0.10 − 0.06 − 0.06 − 0.08 − 0.06 − 0.08 − 0.06 − 0.01 − 0.07	− 0.17 − 0.15 − 0.14 − 0.23 − 0.20 − 0.16 − 0.12 − 0.08 − 0.13	− 0.20 − 0.19 − 0.18 − 0.30 − 0.27 − 0.20 − 0.15 − 0.11 − 0.17	− 0.13 − 0.12 − 0.11 − 0.15 − 0.14 − 0.12 − 0.09 − 0.05 − 0.08

Concentration index (CI), lower (LCI), and upper (UCI) 95% confidence interval data for death ratio, stunting, underweight, and combined health outcomes with subgroup analysis for low (L), lower-middle (LM), and upper middle (UM) country-income level and pre-1995, 1995–2004, and post-2005 survey years.

**Table 2:** Meta-analysis of aggregated RII results.

	Death ratio			Stunting			Underweight			Combined		
	RII	LCI	UCI	RII	LCI	UCI	RII	LCI	UCI	RII	LCI	UCI
Consumption Hybrid Income	2.40 2.75 2.41	1.98 2.19 1.96	2.91 3.46 2.96	1.87 2.04 1.79	1.48 1.59 1.53	2.35 2.60 2.10	1.91 2.34 2.13	1.50 1.82 1.66	2.43 3.01 2.74	2.01 2.32 2.03	1.75 2.01 1.82	2.31 2.69 2.28
L Consumption L Hybrid L Income LM Consumption LM Hybrid LM Income UM Consumption UM Hybrid UM Income	1.81 1.84 1.85 3.12 3.51 2.54 3.54 4.62 3.08	1.55 1.42 1.50 2.30 2.54 1.85 2.79 2.56 1.67	2.10 2.38 2.29 4.23 4.83 3.49 4.50 8.36 5.68	1.47 1.61 1.39 2.07 2.16 1.79 3.49 4.02 2.64	1.30 1.36 1.27 1.31 1.34 1.42 2.01 1.82 2.34	1.66 1.91 1.52 3.26 3.50 2.25 6.07 8.87 2.97	1.52 1.75 1.48 2.14 2.70 2.30 3.56 5.28 3.38	1.22 1.45 1.10 1.30 1.61 1.53 1.14 1.22 1.94	1.89 2.12 2.01 3.51 4.54 3.46 11.10 22.85 5.89	1.53 1.71 1.47 2.34 2.68 2.11 3.45 4.54 2.86	1.39 1.53 1.34 1.79 2.03 1.77 2.54 2.93 2.42	1.69 1.90 1.62 3.07 3.55 2.50 4.70 7.05 3.38
Pre-1995 Consumption Pre-1995 Hybrid Pre-1995 Income 1995–2004 Consumption 1995–2004 Hybrid 1995–2004 Income Post-2005 Consumption Post-2005 Hybrid Post-2005 Income	2.48 2.67 2.28 3.02 4.01 2.81 1.92 2.01 2.05	1.83 2.04 1.77 1.75 2.29 1.72 1.57 1.37 1.27	3.37 3.50 2.94 5.24 7.03 4.60 2.36 2.96 3.30	2.04 1.86 1.88 2.67 2.91 1.76 1.33 1.65 1.65	1.45 1.36 1.40 1.52 1.48 1.35 1.06 1.26 1.18	2.88 2.55 2.52 4.71 5.76 2.29 1.67 2.16 2.32	1.91 2.20 1.92 2.96 3.65 2.45 1.40 1.77 2.17	1.38 1.60 1.30 1.43 1.61 1.53 1.07 1.34 1.50	2.64 3.04 2.85 6.11 8.28 3.93 1.83 2.34 3.14	2.12 2.19 2.00 2.85 3.44 2.17 1.45 1.76 1.79	1.75 1.84 1.68 2.04 2.32 1.78 1.25 1.49 1.50	2.56 2.61 2.38 3.99 5.10 2.65 1.69 2.08 2.13

Relative index of inequality (RII), lower (LCI), and upper (UCI) 95% confidence interval data for death ratio, stunting, underweight, and combined health outcomes with subgroup analysis for low (L), lower-middle (LM), and upper middle (UM) country-income level and pre-1995, 1995–2004, and post-2005 survey years.

**Table 3:** Meta-analysis of aggregate SII results.

	Death ratio			Stunting			Underweight			Combined		
	SII	LCI	UCI	SII	LCI	UCI	SII	LCI	UCI	SII	LCI	UCI
Consumption Hybrid Income	− 0.06 − 0.06 − 0.06	− 0.07 − 0.08 − 0.08	− 0.04 − 0.05 − 0.04	− 0.16 − 0.18 − 0.17	− 0.22 − 0.25 − 0.23	− 0.09 − 0.11 − 0.12	− 0.07 − 0.09 − 0.09	− 0.10 − 0.12 − 0.11	− 0.04 − 0.06 − 0.06	− 0.10 − 0.12 − 0.11	− 0.12 − 0.14 − 0.13	− 0.07 − 0.09 − 0.08
L Consumption L Hybrid L Income LM Consumption LM Hybrid LM Income UM Consumption UM Hybrid UM Income	− 0.05 − 0.05 − 0.05 − 0.06 − 0.07 − 0.06 − 0.07 − 0.07 − 0.06	− 0.07 − 0.08 − 0.09 − 0.08 − 0.09 − 0.08 − 0.15 − 0.12 − 0.12	− 0.03 − 0.02 − 0.02 − 0.04 − 0.05 − 0.04 0.01 − 0.03 − 0.01	− 0.12 − 0.15 − 0.11 − 0.15 − 0.17 − 0.20 − 0.34 − 0.32 − 0.25	− 0.15 − 0.21 − 0.15 − 0.30 − 0.32 − 0.31 − 0.49 − 0.49 − 0.31	− 0.09 − 0.10 − 0.06 − 0.01 − 0.01 − 0.09 − 0.18 − 0.15 − 0.20	− 0.06 − 0.09 − 0.06 − 0.06 − 0.09 − 0.10 − 0.10 − 0.10 − 0.10	− 0.10 − 0.13 − 0.11 − 0.13 − 0.16 − 0.16 − 0.12 − 0.12 − 0.12	− 0.03 − 0.05 0.00 0.00 − 0.01 − 0.05 − 0.08 − 0.08 − 0.07	− 0.08 − 0.10 − 0.07 − 0.09 − 0.11 − 0.13 − 0.17 − 0.16 − 0.13	− 0.10 − 0.13 − 0.10 − 0.14 − 0.16 − 0.17 − 0.25 − 0.22 − 0.18	− 0.06 − 0.07 − 0.04 − 0.05 − 0.07 − 0.08 − 0.08 − 0.10 − 0.08
Pre-1995 Consumption Pre-1995 Hybrid Pre-1995 Income 1995–2004 Consumption 1995–2004 Hybrid 1995–2004 Income Post-2005 Consumption Post-2005 Hybrid Post-2005 Income	− 0.09 − 0.10 − 0.09 − 0.03 − 0.04 − 0.04 − 0.05 − 0.05 − 0.07	− 0.11 − 0.12 − 0.10 − 0.05 − 0.06 − 0.06 − 0.06 − 0.08 − 0.12	− 0.07 − 0.07 − 0.07 − 0.02 − 0.03 − 0.02 − 0.03 − 0.02 − 0.03	− 0.19 − 0.20 − 0.17 − 0.24 − 0.21 − 0.19 − 0.08 − 0.15 − 0.14	− 0.29 − 0.32 − 0.26 − 0.39 − 0.37 − 0.28 − 0.14 − 0.22 − 0.26	− 0.10 − 0.07 − 0.08 − 0.08 − 0.04 − 0.11 − 0.01 − 0.07 − 0.02?	− 0.09 − 0.11 − 0.09 − 0.08 − 0.08 − 0.09 − 0.04 − 0.08 − 0.07	− 0.13 − 0.17 − 0.14 − 0.16 − 0.15 − 0.13 − 0.07 − 0.11 − 0.14	− 0.05 − 0.06 − 0.04 0.00 − 0.01 − 0.04 − 0.01 − 0.05 − 0.01	− 0.12 − 0.14 − 0.11 − 0.11 − 0.11 − 0.11 − 0.05 − 0.10 − 0.09	− 0.16 − 0.18 − 0.15 − 0.17 − 0.16 − 0.14 − 0.08 − 0.13 − 0.13	− 0.09 − 0.09 − 0.08 − 0.06 − 0.06 − 0.07 − 0.03 − 0.07 − 0.04

Slope index of inequality (SII), lower (LCI), and upper (UCI) 95% confidence interval data for death ratio, stunting, underweight, and combined health outcomes with subgroup analysis for low (L), lower-middle (LM), and upper middle (UM) country-income level and pre-1995, 1995–2004, and post-2005 survey years.

Next, inequality measures were broken down by country-income level and by survey year (Tables 1, 2, 3). Concentration index values clearly increase as countries become richer. Asset indices again result in higher concentration index values in 11 out of 12 instances, although none of the differences are statistically significant. While there are no clear secular changes in concentration index levels, magnitudes are higher in the 1995–2004 era. Yet again, there are no statistically different index values, but a suggestive trend emerges, with asset indices resulting in the largest values in seven out of eight comparisons prior to 2005, but income resulting in the largest magnitudes in all four post-2005 comparisons.

The clear trend of higher inequalities among richer countries is replicated with RII measures. There are no significant differences between SES measures, and the hybrid income proxy results in the highest inequality levels in 11 of 12 comparisons. Over time, RII values are highest in the 1995–2004 era and appear to have decreased after 2005. Once again, a suggestive trend emerges with the hybrid income proxy resulting in the largest values in seven out of eight comparisons prior to 2005, but income resulting in the largest magnitudes in all four post-2005 comparisons.

Finally, SII magnitudes are also largest in higher-income countries (albeit with smaller differences than concentration indices and RIIs), but the dominant SES measures change depending on country-income level. The largest SII magnitudes in low-income countries result from the hybrid income proxy in all four cases, but income results in the largest magnitudes in three of four cases for low-middle income countries and consumption in for three of four cases in upper-middle income countries. The SII points to generally decreasing absolute inequality over time, and there is no clear time trend mediating SES measures and inequality magnitudes.

## Discussion

This first systematic empirical investigation of the effect of SES measures on health inequality magnitudes suggests that the use of asset indices, and the hybrid income proxy based on them, may result in larger magnitudes of wealth-related inequality than either household consumption or income. These results are tenuous because the relatively small number of studies available for analysis result in wide confidence intervals, but the fact that asset indices (and hybrid income proxy) result in the largest magnitudes of health inequalities for all 12 overall outcomes, as well as most country-income and survey year subgroupings, is strongly indicative of a true underlying difference. Thinking through these results using the lens of risk may allow for even stronger interpretation. A global health researcher faced with the risk that a change in SES measure could result in a concentration index jumping from -0.07 to -0.19 for stunting in Ghana or from 0.03 to -0.14 for underweight in Nigeria would surely conclude that there is a real risk that SES measures can have a large impact on the magnitudes of health inequalities.

Although there is no clear time trend for health inequality magnitudes postulated by other authors^27^, the findings do replicate results suggesting larger magnitudes of health inequality as country-income levels increase^23^. In theory, asset indices may be more appropriate measures of household SES in lower income countries because a greater percentage of survey respondents receive no regular income and consumption patterns may be more irregular and difficult to measure^5,8^. The results do not provide clear evidence to support this theory, although SII magnitudes were largest in magnitude with the hybrid income proxy (which is based on the asset index) for low-income countries only. This study also finds mixed evidence supporting the assertion that any of the three SES measure is producing larger magnitudes of social health inequality as time goes on. Although larger concentration index and RII values shifted from asset indices and hybrid income proxy to household income in more recent survey years, SII trends are inconclusive. If future research is able to demonstrate continued reductions in the magnitude of health inequalities based on asset indices—as compared to consumption and income—analysts may need to evaluate the degree to which the standard basket of household assets should be revised in light of newly emerging asset classes, such as smartphones^17^.

Differences between SES measures according to health outcomes did emerge, with more statistically significant differences between SES measures for stunting and underweight than child deaths. This may be a statistical effect driven by higher prevalence levels of these outcomes affecting magnitudes of difference between SES measures, or it may be indicative of differences between permanent and transitory household SES levels affecting shorter-term and longer-term health outcomes differently. Regardless of the cause, analysts should be aware of the higher risk for these SES measure-driven differences to emerge when studying these outcomes.

Lastly, the similarities between absolute and relative inequality measures reveal two important findings. First, smaller differences in absolute inequalities between lower and higher-income countries than of relative health inequalities may be due to lower overall disease prevalence along the entire SES spectrum in the richer countries, with greater reductions among higher earners within richer countries^32^. Second, the finding that, just like the asset indices they are based on, the hybrid income proxy method results in larger inequality measures than income or consumption in most of the cases observed provides evidence that this new method may perform more similarly to asset indices than the household incomes they are supposed to simulate^19,20^. Researchers using the new method should recognize that although the absolute scale of predicted income appears similar to actual household income, the underlying SES construct it is measuring may be closer to that measured by asset indices.

A major strength of this study is that it is the first systematic comparison of income, consumption, and asset indices on magnitudes of health inequalities using the same microdata for several countries. While compiling disparate estimations of inequality magnitudes can be suggestive^24^, systematic analysis of microdata is the only way to overcome the uncertainty associated with these efforts. This study also compared only health outcomes that are directly measured and comparable across countries and survey years, and every survey used in analysis is a standardized LSMS. In addition, this is the first study to incorporate the new hybrid income proxy measure to investigate the impact of SES measures on health inequality magnitudes.

However, by limiting the study to these directly comparable household surveys containing income, consumption, and assets, the size of the compiled dataset was effectively limited to a small number of studies. If more surveys were included for analysis, either by obtaining non-public surveys or by analyzing surveys that did not contain household income or consumption modules, the suggestive differences observed may have become statistically significant at the 95% confidence level. The study was also limited by small sample sizes reporting household income in some survey waves, but this is a limitation inherent in the collection of income data from low-income countries motivating many to use consumption or asset measures rather than income data. It is also possible that the countries sampled by the World Bank or the countries that chose to include health modules may be systematically different than countries sampled for DHS or MICS surveys.

These results matter for global health researchers, for multilateral organizations, and for development economists. Global health researchers should be cognizant that their choice of SES measure is not innocuous, as choosing an asset index over household income may represent a more permanent marker of household SES and may result in larger measured magnitudes of inequality. Although this study takes no normative stance on the presence of larger inequalities being a marker of a superior SES measure, the mere presence of difference must be acknowledged and accounted for. This is why multilateral organizations such as the World Bank, USAID, and UNICEF should investigate the effects of their preferred SES measures more thoroughly and inform country partners and researchers of the implications of their choices. Finally, development economists should begin studying the pathways linking SES, whether it is measured using income, consumption, or household assets, with health outcomes. If magnitudes of social health inequality are systematically different depending on the choice of household SES measure, there must be a causal link mediating SES and health through one or more pathways that have yet to be fully identified. There is clearly more research to be done with a more comprehensive set of household surveys and health outcomes, but the apparent increase in the magnitude of health inequalities with the use of asset indices to measure household SES suggests that income, consumption, and asset indices are not as equal as commonly assumed.

## Methods

The largest internationally standardized survey of household living standards is the World Bank’s LSMS, which has been conducted over 100 times in LMICs around the world^31^. Due to their focus on living standards, some, but not all, of these studies capture data on household income, consumption, and/or assets; a feature which is not present in DHS or MICS. Additionally, a subset of LSMS surveys capture information on select health outcomes, although these are limited in number compared to health-focused surveys. This study used the World Bank’s LSMS Dataset Finder^34^ to identify every survey that contained each of income, consumption data, household assets, and data on either (1) child deaths, or (2) child anthropometrics. These outcomes were selected because they were the most commonly available measures in LSMS surveys, can be easily and objectively compared across borders, and are widely accepted as sensitive indicators of the population’s health as a whole^35–37^.

Inclusion criteria limited the search to any nationally representative LSMS with all three household SES measures and at least two health outcomes conducted from inception to 2014. Surveys conducted after 2014 were excluded because the country- and year-specific simulated income distributions needed to calculate the hybrid income proxy have not been updated since their initial release^20^. A maximum of two survey years per country were included, in order of most recently conducted survey, to prevent overrepresentation of select countries. Only surveys that contained pre-calculated household consumption and income modules and were available for public use were compiled for this study (i.e., no special permission needed from a country statistical agency). The data search was originally conducted in April 2018 and subsequently updated in August 2022, with no new datasets meeting inclusion criteria found in the most recent search.

Every survey was closely examined to ensure direct comparability, and data from each of 22 surveys summarized in Table 4 was compiled. Each health measure was calculated by the author from raw height, weight, age, and fertility data, even if the survey already included precalculated variables, in order to ensure comparability. Child deaths and births for women aged 15–49 were included for analysis to replicate commonly used DHS methods^38^, and a simple ratio of deaths to births was used as a proxy for infant mortality. No survey weights were used because the outcome of interest is the household SES measure itself, not population-level health or SES estimates. Mortality ratios could not be calculated for Nigeria because a births per woman variable was not present in LSMS data. Child nutrition outcomes of stunting and underweight were calculated as being more than two standard deviations from the 2006 WHO child growth standards for height and weight for age (in months) using Stata zscore06 package for all children under the age of five^39,40^.

**Table 4:** Living Standards Measurement Survey (LSMS) characteristics.

Survey Code	Country	Year	Income group	Survey name	Primary investigators	Sample size
ALB_2002	Albania	2002	Low-Middle	Living Standards Measurement Survey 2002 (Wave 1 Panel)	Institute of Statistics of Albania	3,599
BRA_1996	Brazil	1996	Upper-Middle	Living Standards Measurement Study Survey 1996–1997	Instituto Brasileiro de Geografia e Estatísticai / Brazilian Geographical and Statistical institute (IBGE)	4,940
CIV_1987	Cote d'Ivoire	1987	Low-Middle	Enquête Permanente Auprès des Ménages 1987–1988 (Wave 3 Panel)	Direction de la Statistique—Ministère de l'Economie et des Finances	1,600
CIV_1988	Cote d'Ivoire	1988	Low-Middle	Enquête Permanente Auprès des Ménages 1988–1989 (Wave 4 Panel)	Direction de la Statistique—Ministère de l'Economie et des Finances	1,600
GHA_1988	Ghana	1988	Low	Living Standards Survey II 1988–1989	Ghana Statistical Service (GSS)	3,104
GHA_2009	Ghana	2009	Low	Socioeconomic Panel Survey: 2009–2010	Institute of Statistical, Social and Economic Research—University of Ghana	4,972
GTM_2000	Guatemala	2000	Low-Middle	Encuesta Nacional sobre Condiciones de Vida 2000	Instituto Nacional de Estadística (INE)	7,276
GUY_1992	Guyana	1992	Low	Living Standards Measurements Survey 1992	Bureau of Statistics—Ministry of Finance	1,818
KGZ_1997	Kyrgyzstan	1997	Low	Poverty Monitoring Survey 1997	National Statistical Committee (NATSTATCOM)	2,876
KGZ_1998	Kyrgyzstan	1998	Low	Poverty Monitoring Survey 1998	National Statistical Committee (NATSTATCOM)	2,979
NGA_2010	Nigeria	2010	Low	General Household Survey, Panel 2010	National Bureau of Statistics (NBS)—Federal Government of Nigeria (FGN)	4,935
NGA_2012	Nigeria	2012	Low	General Household Survey, Panel 2012–2013	National Bureau of Statistics (NBS)—Federal Government of Nigeria (FGN)	4,814
PAK_1991	Pakistan	1991	Low	Integrated Household Survey 1991	Federal Bureau of Statistics (FBS)	4,799
PAN_1997	Panama	1997	Low-Middle	Encuesta de Niveles de Vida 1997	Ministerio de Planificacion y Politica Economica	4,945
PAN_2003	Panama	2003	Upper-Middle	Encuesta de Niveles de Vida 2003	Ministerio de Economía y Finanzas (MEF)	6,363
PER_1994	Peru	1994	Low-Middle	Encuesta Nacional de Hogares sobre Medición de Niveles de Vida 1994	Instituto Nacional de Estadística (INE)	3,623
TJK_2007	Tajikistan	2007	Low	Living Standards Survey 2007	State Statistical Agency	4,860
TLS_2007	Timor-Leste	2007	Low-Middle	Survey of Living Standards 2007 and Extension 2008	National Statistics Directorate	4,477
TZA_2010	Tanzania	2010	Low	National Panel Survey 2010–2011	National Bureau of Statistics—Ministry of Finance, Tanzania	3,924
UGA_2011	Uganda	2011	Low	National Panel Survey 2011–2012	Uganda Bureau of Statistics (UBOS)—Ministry of Finance, Planning and Economic Development	2,837
UGA_2013	Uganda	2013	Low	National Panel Survey 2013–2014	Uganda Bureau of Statistics—Government of Uganda	3,119
ZAF_1993	South Africa	1993	Upper-Middle	Integrated Household Survey 1993	Southern Africa Labour and Development Research Unit	8,810

Sample size refers to the number of households containing information on at least one of income, consumption, or asset index. Samples may differ for each health outcome or individual SES measure.

Household income and consumption data were extracted directly from their respective modules and kept in local currencies, since all health inequality calculations were conducted within single survey country-years. Although some researchers choose to use per capita or equivalized household income and consumption^1,23^, aggregate household income and consumption were selected for this study. Aggregate household income and consumption are more directly comparable to the dimension of SES measured by the asset index because the unit of measurement is the household rather than the individual, and household SES is more stable due to intra-household income or consumption substitution in the case of income shocks^6,41^.

Asset indices were then calculated using Kolenikov and Angeles’^12^ update of Filmer and Pritchett’s^9^ original PCA methods. This method was chosen because a review of the literature on asset index construction using household surveys identified polychoric PCA as a more statistically appropriate, easily calculated, and reliable indicator of household SES^21^. Despite these advantages, the two methods are almost always highly correlated (Spearman correlation coefficient > 0.90). Every household commodity in each survey wave was included for calculation of the asset indices, resulting in a range of 10 to 37 assets per survey. Finally, a “hybrid” income proxy method was used to map an estimated household income to each household’s relative ranking of asset index wealth centiles for the survey’s country-year. This was done by dividing each survey’s households into 100 equal parts according to the relative ranking determined by the asset index, and then assigning each of those centiles a predicted income according to the year and country in which the survey was conducted using an open-access dataset developed for this purpose^20,42^. Summary statistics of all health outcomes are presented in Table 5.

**Table 5:** Summary of health outcome prevalence for all LSMS included for analysis.

Survey code	Country	Year	Mean child death ratio	Stunting prevalence	Underweight prevalence
ALB_2002	Albania	2002	0.03	0.43	0.10
BRA_1996	Brazil	1996	0.04	0.20	0.06
CIV_1987	Cote d'Ivoire	1987	0.12	0.18	0.10
CIV_1988	Cote d'Ivoire	1988	0.12	0.19	0.11
GHA_1988	Ghana	1988	0.14	0.34	0.22
GHA_2009	Ghana	2009	0.06	0.29	0.22
GTM_2000	Guatemala	2000	0.07	0.50	0.17
GUY_1992	Guyana	1992	0.07	0.14	0.13
KGZ_1997	Kyrgyzstan	1997		0.37	0.10
KGZ_1998	Kyrgyzstan	1998	0.03	0.41	0.14
NGA_2010	Nigeria	2010		0.37	0.28
NGA_2012	Nigeria	2012		0.21	0.11
PAK_1991	Pakistan	1991	0.13	0.44	0.38
PAN_1997	Panama	1997	0.02	0.20	0.06
PAN_2003	Panama	2003	0.03	0.29	0.06
PER_1994	Peru	1994	0.06	0.34	0.09
TJK_2007	Tajikistan	2007	0.06	0.38	0.15
TLS_2007	Timor-Leste	2007	0.07	0.45	0.42
TZA_2010	Tanzania	2010		0.32	0.13
UGA_2011	Uganda	2011	0.11	0.21	0.08
UGA_2013	Uganda	2013	0.09	0.21	0.07
ZAF_1993	South Africa	1993	0.10	0.26	0.13

Outcomes are not population weighted and are therefore not indicators of population-level health.

Wealth-related inequalities in health outcomes were then calculated using a national poverty line proxy, the concentration index, RII, and SII. For illustrative purposes, a simple proxy of national poverty lines was created by identifying the lowest quintile of households ordered by assets, consumption, and income. This approach reflects the fact that national poverty lines commonly result in approximately 20% of the population being classified as poor^43^, but it does not take into account health outcomes for the remaining 80% of the population. The value of the concentration index corresponds to two times the area between the line of equality and the concentration curve, which can be interpreted as the percentage of the total outcome of interest that would have to be redistributed from the richest half to the poorest half of the population. It is worth noting that concentration index magnitudes will not change if health and rank difference do not covary, because even if the relative ranking of households changes, a lack of correlation with health would result in no change to the index value^22^. O’Donnell et al^44^. *conindex* command based on the convenient regression method was used to calculate index values and standard errors, with Wagstaff corrections for bounded health outcome of stunting, underweight, and ratio of child deaths^31,45,46^. Concentration index values were calculated for income, consumption, and asset index values as measures of SES, but the hybrid income proxy method was not used because household orderings are based entirely on asset index values, meaning there would be no difference except for loss of granularity after converting to centiles.

The SII can be interpreted as the absolute difference in health outcome between the richest and poorest household, taking into account the entire distribution of households in a regression, while the RII is a ratio measure of the prevalence of the health outcome of the poorest households compared to the richest households, again taking the entire distribution of households into account via regression^47,48^ These health inequality measures were calculated using a generalized linear model (GLM) with a logarithmic link for RIIs and an identity link for SIIs using methods described by Ernstsen et al^49^. Household rankings for these methods use ridits of income, consumption, and hybrid income proxy calculated using methods described by Bross^50^, meaning that the scale of differences in household wealth is taken into account. Unlike the concentration index, the hybrid income proxy was used in lieu of the asset index, because both the SII and RII require an interval rather than ordinal SES measure. SIIs could not be calculated for Panama 1997 and Peru 1994 (underweight only) because GLM models would not converge. Taken together, these three metrics measure scale-independent SES against relative health inequality (concentration index), scale dependent SES against relative health inequality (RII), and scale dependent SES against absolute health inequality (SII).

In addition to presenting survey-by-survey results, results were aggregated using meta-analytic techniques to produce a single measure of association. The pooling of aggregate data was conducted using a random-effects inverse-variance model with DerSimonian-Laird estimate of tau^2^. Each inequality estimate was aggregated by survey wave with standard errors and analyzed using random effects for generic effect measures with the *metan* command^51,52^, and repeated for subgroups of survey year (pre-1995, 1995–2004, and post-2004), outcome (child deaths, stunting, and underweight), and World Bank country-income group (low, low-middle, and upper-middle). Overall effect sizes, as well as inequality magnitudes of each subgrouping, were then examined for significant differences between the three major SES measurement methods of income, consumption, and asset indices (or income proxy hybrid in the case of RII and SII).

### Supplementary Information

Supplementary Information.

## Data Availability

The Living Standards Measurement Study (LSMS) datasets used to conduct this study are all publicly available through the World Bank’s Microdata Library: https://microdata.worldbank.org/index.php/home.
