# Supplementary material for: Systematic comparison of household income, consumption, and assets to measure health inequalities in low- and middle-income countries
Source: Sci Rep. 2024 Feb 15;14:3851. doi: 10.1038/s41598-024-54170-1 (PMC10869835; doi:10.1038/s41598-024-54170-1)

Supplementary Information

Systematic Comparison of Household Income, Consumption, and Assets to Measure Health Inequalities in Low- and Middle-Income Countries

Mathieu J.P. Poirier

Table S1. Survey and quintile-specific observations, sex ratio, and prevalence for stunting (ST) and underweight (UW) using income, consumption, and assets

	Income					Consumption					Assets				
	N	Female	Age	ST	UW	N	Sex	Age	ST	UW	N	Sex	Age	ST	UW
ALB_2002															
1	335	0.430	32.6	0.442	0.125	279	0.466	32.9	0.391	0.115	367	0.493	32.6	0.460	0.106
2	221	0.439	29.9	0.516	0.154	263	0.452	32.2	0.490	0.106	312	0.429	31.1	0.429	0.122
3	308	0.458	31.1	0.435	0.078	260	0.458	31.0	0.469	0.100	246	0.419	31.7	0.382	0.069
4	222	0.446	31.7	0.428	0.117	251	0.406	30.6	0.414	0.120	191	0.476	29.8	0.435	0.110
5	252	0.413	30.5	0.361	0.052	285	0.404	29.6	0.414	0.081	217	0.346	30.2	0.456	0.111
BRA_1996															
1	435	0.485	30.1	0.301	0.092						560	0.473	29.8	0.293	0.089
2	397	0.511	31.0	0.176	0.065						419	0.549	30.2	0.189	0.057
3	332	0.509	30.4	0.181	0.045						344	0.445	31.6	0.163	0.044
4	276	0.536	31.5	0.167	0.029						244	0.553	30.8	0.164	0.029
5	207	0.507	30.3	0.116	0.024						195	0.533	32.8	0.108	0.031
CIV_1987															
1	249	0.498	31.4	0.233	0.104	201	0.547	31.3	0.244	0.090	426	0.472	31.8	0.228	0.131
2	345	0.493	32.3	0.214	0.099	370	0.484	31.1	0.224	0.119	496	0.484	31.7	0.179	0.119
3	479	0.480	31.5	0.188	0.115	505	0.487	32.5	0.208	0.119	484	0.488	32.2	0.155	0.085
4	559	0.487	32.4	0.174	0.131	593	0.464	32.9	0.174	0.106	447	0.494	32.9	0.177	0.087
5	571	0.504	33.9	0.135	0.068	564	0.509	33.5	0.112	0.085	353	0.518	34.2	0.164	0.091
CIV_1988															
1	233	0.459	29.2	0.240	0.150	200	0.470	28.7	0.290	0.140	387	0.463	30.9	0.204	0.124
2	369	0.496	32.3	0.203	0.106	352	0.494	31.6	0.216	0.122	467	0.450	31.8	0.216	0.124
3	417	0.520	32.4	0.201	0.098	467	0.505	32.3	0.203	0.111	459	0.510	33.5	0.170	0.118
4	537	0.443	33.0	0.196	0.102	526	0.475	33.3	0.173	0.089	448	0.520	32.7	0.172	0.078
5	549	0.519	34.5	0.149	0.100	576	0.497	34.9	0.148	0.101	356	0.511	35.2	0.194	0.090
GHA_1988															
1						365	0.493	33.4	0.362	0.230	655	0.501	32.8	0.389	0.263
2						511	0.503	30.6	0.348	0.233	603	0.514	33.1	0.443	0.274
3						603	0.493	33.7	0.406	0.250	678	0.509	33.6	0.382	0.258
4						686	0.520	33.2	0.340	0.206	549	0.486	32.8	0.279	0.166
5						741	0.495	34.1	0.286	0.201	413	0.492	33.2	0.157	0.099
GHA_2009															
1						414	0.478	34.5	0.297	0.232	926	0.524	34.3	0.362	0.241
2						625	0.522	34.2	0.301	0.246	545	0.475	34.1	0.301	0.251
3						678	0.501	34.2	0.326	0.230	416	0.466	34.5	0.252	0.216
4						618	0.494	35.0	0.270	0.223	378	0.513	34.7	0.238	0.169
5						596	0.503	35.0	0.233	0.168	342	0.494	34.9	0.173	0.164
GTM_2000															
1	1,205	0.491	29.0	0.589	0.222	1,192	0.484	29.3	0.637	0.236	1559	0.509	29.3	0.604	0.227
2	1,318	0.489	29.9	0.581	0.211	1,391	0.494	29.8	0.578	0.223	1392	0.481	29.3	0.585	0.224
3	1,271	0.500	30.3	0.496	0.164	1,272	0.501	29.9	0.499	0.153	1188	0.481	30.8	0.536	0.166
4	1,088	0.497	30.2	0.427	0.136	1,117	0.498	29.8	0.415	0.136	941	0.514	30.5	0.336	0.090
5	819	0.490	29.4	0.309	0.078	771	0.490	30.4	0.248	0.053	663	0.477	29.3	0.216	0.045
GUY_1992															
1	113	0.446	31.4	0.150	0.124	94	0.478	30.7	0.170	0.117	120	0.470	29.4	0.133	0.150
2	114	0.450	29.6	0.149	0.140	126	0.448	31.6	0.151	0.183	133	0.447	29.5	0.113	0.120
3	131	0.473	29.6	0.130	0.130	111	0.445	30.6	0.162	0.144	128	0.445	28.9	0.172	0.141
4	118	0.436	30.8	0.161	0.144	149	0.514	28.9	0.148	0.121	122	0.508	32.6	0.156	0.115
5	125	0.524	29.7	0.120	0.096	121	0.442	29.6	0.083	0.066	98	0.469	31.0	0.133	0.102
KGZ_1997															
1	154	0.468	38.0	0.390	0.143	140	0.493	40.4	0.436	0.150	320	0.500	38.3	0.338	0.106
2	181	0.486	37.3	0.398	0.122	233	0.468	36.7	0.421	0.124	294	0.476	38.4	0.395	0.068
3	243	0.535	39.2	0.391	0.111	246	0.476	38.0	0.431	0.093	237	0.511	37.9	0.435	0.122
4	293	0.485	38.5	0.365	0.082	250	0.512	37.5	0.348	0.084	164	0.512	37.8	0.372	0.116
5	291	0.491	36.7	0.326	0.082	294	0.517	38.3	0.272	0.085	98	0.500	36.8	0.255	0.122
KGZ_1998															

	Income					Consumption					Assets				
	N	Female	Age	ST	UW	N	Sex	Age	ST	UW	N	Sex	Age	ST	UW
1	283	0.516	36.0	0.466	0.152	294	0.432	37.4	0.459	0.095	506	0.471	35.3	0.433	0.140
2	338	0.501	36.8	0.408	0.121	378	0.525	36.9	0.452	0.159	433	0.519	37.8	0.397	0.150
3	304	0.510	38.1	0.431	0.138	337	0.497	35.5	0.409	0.145	286	0.489	37.4	0.434	0.154
4	361	0.457	36.6	0.391	0.133	384	0.505	37.0	0.370	0.143	275	0.467	37.1	0.433	0.131
5	403	0.483	35.6	0.385	0.159	334	0.467	36.4	0.377	0.150	143	0.486	35.5	0.350	0.119
NGA_2010															
1	62	0.463	33.4	0.484	0.258	326	0.460	32.3	0.377	0.218	502	0.482	31.6	0.416	0.315
2	67	0.524	31.9	0.507	0.418	512	0.508	31.4	0.422	0.285	530	0.435	31.7	0.415	0.330
3	56	0.439	29.2	0.321	0.196	582	0.467	31.4	0.368	0.313	495	0.490	31.1	0.343	0.267
4	55	0.438	28.1	0.364	0.164	578	0.498	30.4	0.315	0.285	492	0.491	31.1	0.333	0.260
5	42	0.622	28.3	0.095	0.095	578	0.459	31.5	0.370	0.277	385	0.459	30.4	0.242	0.174
NGA_2012															
1	43	0.442	30.6	0.140	0.093	297	0.448	30.8	0.249	0.128	673	0.478	31.4	0.218	0.123
2	44	0.477	29.3	0.273	0.023	556	0.496	32.0	0.218	0.133	597	0.461	31.6	0.261	0.127
3	59	0.593	28.8	0.220	0.153	643	0.474	30.2	0.215	0.112	498	0.504	30.1	0.183	0.106
4	22	0.455	33.8	0.182	0.091	635	0.490	31.5	0.198	0.096	537	0.471	31.1	0.214	0.110
5	37	0.486	34.2	0.189	0.054	606	0.474	31.6	0.216	0.111	461	0.497	31.0	0.174	0.082
PAK_1991															
1	685	0.512	29.8	0.463	0.413	618	0.518	29.9	0.500	0.437	759	0.503	29.3	0.478	0.480
2	761	0.524	29.2	0.498	0.423	727	0.527	29.9	0.470	0.406	767	0.527	30.2	0.499	0.374
3	775	0.503	30.0	0.489	0.426	768	0.487	29.9	0.445	0.397	879	0.522	30.7	0.504	0.407
4	788	0.509	29.4	0.411	0.340	887	0.516	28.8	0.419	0.370	818	0.484	29.3	0.379	0.339
5	902	0.479	29.1	0.357	0.308	916	0.479	29.2	0.390	0.309	680	0.479	27.5	0.319	0.279
PAN_1997															
1						525	0.488	28.6	0.430	0.120	724	0.453	29.3	0.452	0.127
2						578	0.462	29.3	0.230	0.080	479	0.503	29.4	0.136	0.048
3						434	0.493	28.9	0.113	0.035	440	0.493	26.9	0.077	0.020
4						427	0.492	29.1	0.082	0.014	370	0.503	29.4	0.046	0.016
5						330	0.533	28.7	0.039	0.009	279	0.538	29.3	0.043	0.011
PAN_2003															
1	718	0.506	29.2	0.421	0.102	659	0.496	28.4	0.487	0.106	948	0.487	29.2	0.500	0.117
2	600	0.440	30.3	0.297	0.060	725	0.469	30.0	0.303	0.070	651	0.467	30.0	0.266	0.046
3	609	0.496	28.9	0.284	0.049	569	0.480	29.9	0.257	0.046	563	0.481	29.6	0.179	0.023
4	561	0.480	30.0	0.210	0.037	534	0.466	29.7	0.204	0.030	442	0.466	29.8	0.143	0.025
5	418	0.500	31.8	0.167	0.024	435	0.515	32.0	0.110	0.016	318	0.535	32.1	0.104	0.016
PER_1994															
1	561	0.503	29.3	0.453	0.152	567	0.488	29.6	0.490	0.164	668	0.497	28.9	0.488	0.156
2	542	0.494	30.8	0.415	0.107	559	0.533	29.8	0.435	0.095	568	0.487	30.4	0.451	0.102
3	460	0.525	30.4	0.317	0.061	462	0.463	30.5	0.292	0.063	428	0.507	30.4	0.257	0.047
4	389	0.478	29.8	0.257	0.046	407	0.516	30.9	0.199	0.039	340	0.456	30.7	0.135	0.029
5	344	0.466	29.1	0.148	0.032	301	0.463	28.8	0.130	0.030	224	0.547	30.4	0.098	0.018
TJK_2007															
1						305	0.485	30.7	0.407	0.141	570	0.495	30.7	0.461	0.156
2						430	0.488	32.1	0.393	0.165	539	0.464	30.5	0.425	0.167
3						495	0.491	29.6	0.354	0.123	628	0.467	29.4	0.389	0.156
4						635	0.444	30.2	0.422	0.137	589	0.497	29.3	0.319	0.143
5						836	0.490	28.7	0.360	0.163	368	0.462	30.0	0.299	0.095
TLS_2007															
1						476	0.492	30.2	0.429	0.424	723	0.499	29.1	0.426	0.443
2						756	0.499	29.8	0.413	0.402	777	0.493	30.1	0.425	0.390
3						838	0.505	30.9	0.438	0.427	748	0.489	31.7	0.455	0.421
4						876	0.508	31.4	0.468	0.419	822	0.505	31.2	0.471	0.436
5						991	0.474	29.7	0.474	0.424	867	0.489	30.0	0.459	0.409
TZA_2010															
1						427	0.482	28.0	0.410	0.185	775	0.471	28.3	0.381	0.159
2						568	0.514	28.6	0.375	0.158	738	0.505	28.9	0.364	0.168
3						641	0.487	29.0	0.309	0.122	594	0.517	28.9	0.345	0.133
4						680	0.491	29.1	0.293	0.121	478	0.533	29.2	0.241	0.098

	Income					Consumption					Assets				
	N	Female	Age	ST	UW	N	Sex	Age	ST	UW	N	Sex	Age	ST	UW
5						655	0.530	28.8	0.232	0.105	408	0.522	28.3	0.157	0.074
UGA_2011															
1	455	0.477	29.5	0.222	0.088	362	0.459	29.3	0.224	0.122	544	0.489	29.9	0.276	0.123
2	459	0.519	30.0	0.240	0.105	518	0.500	30.2	0.234	0.097	521	0.482	30.5	0.186	0.063
3	468	0.506	30.5	0.222	0.068	560	0.536	30.1	0.223	0.071	489	0.540	31.4	0.233	0.082
4	426	0.474	31.6	0.192	0.073	551	0.510	30.6	0.211	0.065	506	0.512	30.6	0.194	0.071
5	468	0.553	31.6	0.156	0.043	520	0.542	31.7	0.138	0.040	444	0.541	30.1	0.119	0.034
UGA_2013															
1	57	0.509	30.8	0.211	0.035	419	0.504	27.6	0.232	0.086	619	0.502	30.2	0.236	0.103
2	40	0.375	30.5	0.150	0.025	538	0.517	28.6	0.247	0.087	533	0.527	28.1	0.225	0.081
3	28	0.286	33.8	0.107	0.143	555	0.497	29.7	0.243	0.085	535	0.503	29.3	0.236	0.080
4	28	0.607	31.0	0.071	0.071	562	0.514	29.8	0.181	0.064	496	0.504	29.4	0.214	0.054
5	34	0.706	35.1	0.147	0.000	509	0.519	30.6	0.138	0.051	397	0.514	29.8	0.098	0.038
ZAF_1993															
1	1,099	0.496	29.5	0.334	0.168	953	0.461	30.5	0.366	0.159	1654	0.481	29.9	0.323	0.138
2	1,097	0.491	29.6	0.290	0.129	1,031	0.490	29.1	0.296	0.141	1196	0.493	29.9	0.281	0.165
3	1,020	0.480	30.6	0.282	0.158	1,123	0.508	29.4	0.240	0.136	898	0.510	29.4	0.247	0.131
4	1,036	0.499	30.0	0.212	0.103	1,205	0.496	30.4	0.231	0.118	675	0.505	30.2	0.178	0.089
5	622	0.513	30.4	0.113	0.053	648	0.522	30.7	0.114	0.065	544	0.496	31.0	0.118	0.053

Note: N refers to the number of observations, Female is the proportion of children that are female, age is the mean age in months, ST is the prevalence of stunting, and UW is the prevalence of underweight in each quintile, as measured by income, consumption, and assets

Table S2. Survey and quintile-specific prevalence for births, deaths, and child death ratio using income, consumption, and assets

	Income				Consumption				Assets			
	N	Births	Deaths	Death ratio	N	Births	Deaths	Death ratio	N	Births	Deaths	Death ratio
ALB_2002												
1	517	3.29	0.23	0.048	441	2.87	0.16	0.037	551	3.31	0.24	0.050
2	450	2.94	0.16	0.042	490	3.00	0.18	0.041	558	2.95	0.13	0.030
3	604	2.72	0.11	0.027	549	2.78	0.14	0.034	536	2.70	0.12	0.030
4	518	2.63	0.09	0.022	579	2.73	0.11	0.027	491	2.45	0.08	0.022
5	573	2.26	0.05	0.015	603	2.46	0.06	0.016	519	2.28	0.05	0.017
BRA_1996												
1	541	3.39	0.42	0.085					662	3.94	0.49	0.089
2	609	3.08	0.24	0.047					700	2.99	0.20	0.048
3	626	2.81	0.18	0.042					686	2.57	0.16	0.039
4	624	2.58	0.14	0.033					619	2.48	0.12	0.027
5	620	2.33	0.05	0.014					574	2.13	0.05	0.016
CIV_1987												
1	161	3.81	0.66	0.166	160	3.98	0.83	0.200	187	4.79	0.98	0.184
2	199	4.14	0.63	0.142	211	4.15	0.68	0.148	219	4.38	0.67	0.147
3	214	4.28	0.63	0.118	223	4.38	0.59	0.097	204	4.31	0.70	0.124
4	209	4.31	0.56	0.106	222	3.89	0.50	0.110	208	3.67	0.35	0.093
5	215	3.91	0.47	0.085	199	4.02	0.36	0.060	184	3.23	0.22	0.050
CIV_1988												
1	169	4.04	0.73	0.140	161	4.27	0.86	0.160	207	4.77	0.97	0.177
2	201	4.42	0.70	0.130	211	4.08	0.65	0.139	230	4.85	0.79	0.139
3	226	4.13	0.56	0.125	238	4.56	0.70	0.115	222	4.41	0.70	0.124
4	243	4.41	0.68	0.121	234	4.18	0.58	0.104	214	3.52	0.34	0.093
5	222	4.05	0.47	0.096	223	3.98	0.38	0.100	192	3.36	0.26	0.067
GHA_1988												
1					258	3.79	0.86	0.195	377	4.37	0.84	0.161
2					345	3.69	0.66	0.140	361	4.06	0.79	0.179
3					387	4.02	0.70	0.149	381	4.19	0.74	0.141
4					413	4.06	0.64	0.124	346	3.93	0.53	0.115
5					410	4.12	0.52	0.119	345	3.18	0.38	0.107
GHA_2009												
1					332	3.42	0.30	0.077	695	4.24	0.49	0.092
2					575	3.75	0.34	0.068	491	4.04	0.32	0.061
3					617	3.91	0.36	0.068	441	3.72	0.25	0.049
4					641	3.77	0.34	0.062	463	3.29	0.17	0.035
5					677	3.49	0.20	0.042	442	2.83	0.12	0.031
GTM_2000												
1	1,028	3.92	0.49	0.092	981	3.83	0.51	0.097	1,205	4.67	0.52	0.087
2	1,140	4.33	0.42	0.075	1,161	4.36	0.43	0.076	1,221	4.48	0.47	0.080
3	1,200	4.16	0.39	0.067	1,248	4.21	0.42	0.075	1,220	4.16	0.40	0.069
4	1,268	3.98	0.31	0.053	1,262	4.12	0.29	0.049	1,170	3.41	0.25	0.056
5	1,257	3.31	0.23	0.049	1,277	3.18	0.20	0.042	1,113	2.84	0.14	0.035
GUY_1992												
1	168	3.19	0.35	0.077	136	3.14	0.30	0.072	208	3.32	0.39	0.084
2	187	3.13	0.44	0.092	208	3.04	0.38	0.077	202	2.94	0.28	0.064
3	196	2.66	0.26	0.070	201	2.66	0.26	0.082	185	2.78	0.26	0.064
4	208	2.63	0.24	0.072	214	2.89	0.30	0.068	191	2.65	0.26	0.063
5	195	2.97	0.19	0.041	195	2.87	0.22	0.052	168	2.77	0.26	0.074
KGZ_1998												
1	423	2.94	0.15	0.038	351	2.59	0.10	0.033	559	3.50	0.13	0.031
2	461	3.09	0.16	0.038	485	3.06	0.12	0.032	538	3.32	0.14	0.035
3	460	3.00	0.12	0.033	506	3.15	0.14	0.039	447	3.34	0.20	0.035
4	515	3.13	0.12	0.026	538	3.36	0.14	0.031	455	2.71	0.13	0.038
5	553	3.03	0.10	0.025	589	2.94	0.15	0.031	362	1.90	0.06	0.023

	Income				Consumption				Assets			
	N	Births	Deaths	Death ratio	N	Births	Deaths	Death ratio	N	Births	Deaths	Death ratio
PAK_1991												
1	807	5.03	0.90	0.160	699	4.46	0.90	0.180	879	4.93	0.91	0.164
2	861	5.09	0.91	0.159	837	4.91	0.81	0.143	915	4.93	0.86	0.157
3	898	5.05	0.73	0.123	911	5.34	0.83	0.134	955	5.06	0.85	0.146
4	956	5.07	0.77	0.127	1,025	5.21	0.73	0.115	927	5.07	0.66	0.108
5	1,073	4.43	0.52	0.100	1,130	4.58	0.57	0.107	895	4.56	0.47	0.083
PAN_1997												
1					522	4.18	0.36	0.056	704	4.50	0.35	0.054
2					683	3.58	0.15	0.026	604	3.55	0.11	0.019
3					702	3.11	0.07	0.015	702	2.86	0.06	0.017
4					790	2.83	0.07	0.019	727	2.61	0.06	0.015
5					798	2.41	0.04	0.009	750	2.29	0.03	0.009
PAN_2003												
1	766	3.65	0.17	0.031	664	3.61	0.21	0.045	939	4.08	0.26	0.048
2	846	3.20	0.14	0.030	903	3.34	0.17	0.032	911	3.19	0.10	0.024
3	940	3.13	0.10	0.024	938	3.14	0.10	0.022	913	2.84	0.08	0.020
4	1,035	2.80	0.10	0.028	1,016	2.78	0.06	0.018	919	2.58	0.05	0.016
5	909	2.41	0.05	0.016	1,001	2.43	0.05	0.017	839	2.29	0.05	0.018
PER_1994												
1	551	4.09	0.54	0.094	530	4.14	0.66	0.109	591	4.30	0.59	0.104
2	577	3.92	0.49	0.091	574	3.87	0.43	0.084	604	4.13	0.56	0.094
3	576	3.55	0.35	0.061	599	3.59	0.32	0.055	577	3.49	0.29	0.056
4	625	3.26	0.24	0.046	634	3.27	0.23	0.046	588	3.06	0.14	0.033
5	594	3.00	0.15	0.035	586	2.96	0.14	0.036	482	2.71	0.11	0.032
TJK_2007												
1					645	3.10	0.29	0.082	910	3.81	0.30	0.064
2					827	3.45	0.26	0.059	909	3.61	0.28	0.066
3					912	3.51	0.27	0.065	945	3.52	0.25	0.059
4					954	3.66	0.25	0.054	948	3.18	0.21	0.056
5					1,143	3.45	0.22	0.052	742	3.10	0.21	0.056
TLS_2007												
1					433	3.62	0.53	0.109	595	4.28	0.57	0.101
2					613	4.00	0.48	0.084	605	4.53	0.49	0.080
3					656	4.58	0.46	0.072	625	4.32	0.45	0.073
4					720	4.56	0.39	0.063	689	4.38	0.36	0.060
5					838	4.54	0.35	0.054	746	4.16	0.31	0.055
UGA_2011												
1	286	4.95	0.86	0.136	241	4.90	0.86	0.138	329	4.99	0.81	0.132
2	314	5.19	0.81	0.127	340	4.84	0.79	0.124	328	5.37	0.77	0.110
3	316	4.93	0.68	0.106	380	5.01	0.68	0.111	344	5.35	0.80	0.123
4	333	4.94	0.64	0.104	380	5.06	0.65	0.102	381	4.98	0.65	0.112
5	353	4.61	0.45	0.073	407	4.63	0.42	0.071	371	3.81	0.30	0.060
UGA_2013												
1	59	3.44	0.41	0.124	372	3.61	0.58	0.121	532	4.11	0.59	0.105
2	41	2.59	0.17	0.040	513	3.60	0.49	0.104	526	3.76	0.44	0.094
3	40	2.88	0.18	0.053	569	3.55	0.43	0.093	571	3.83	0.52	0.104
4	56	1.89	0.16	0.065	647	3.83	0.42	0.087	605	3.39	0.38	0.089
5	47	2.55	0.30	0.082	742	2.84	0.26	0.068	605	2.28	0.18	0.068
ZAF_1993												
1	1,349	3.34	0.54	0.132	1,137	3.32	0.65	0.161	1,635	3.55	0.63	0.140
2	1,305	3.18	0.50	0.122	1,326	3.16	0.46	0.118	1,604	3.04	0.41	0.113
3	1,371	3.02	0.39	0.100	1,529	2.96	0.36	0.092	1,370	2.81	0.32	0.086
4	1,597	2.79	0.31	0.083	1,686	2.84	0.29	0.076	1,296	2.59	0.25	0.077
5	1,314	2.45	0.15	0.054	1,373	2.59	0.19	0.056	1,170	2.55	0.19	0.052

Note: N refers to the number of observations, Births is the mean number of births, Deaths is the mean number of deaths, and Death Ratio si the proportion of deaths to births in each quintile, as measured by income, consumption, and assets

Figure S1. Charts of the proportion stunted, proportion underweight, and mean death ratio for the lowest quintile of assets, consumption, and income for each country.

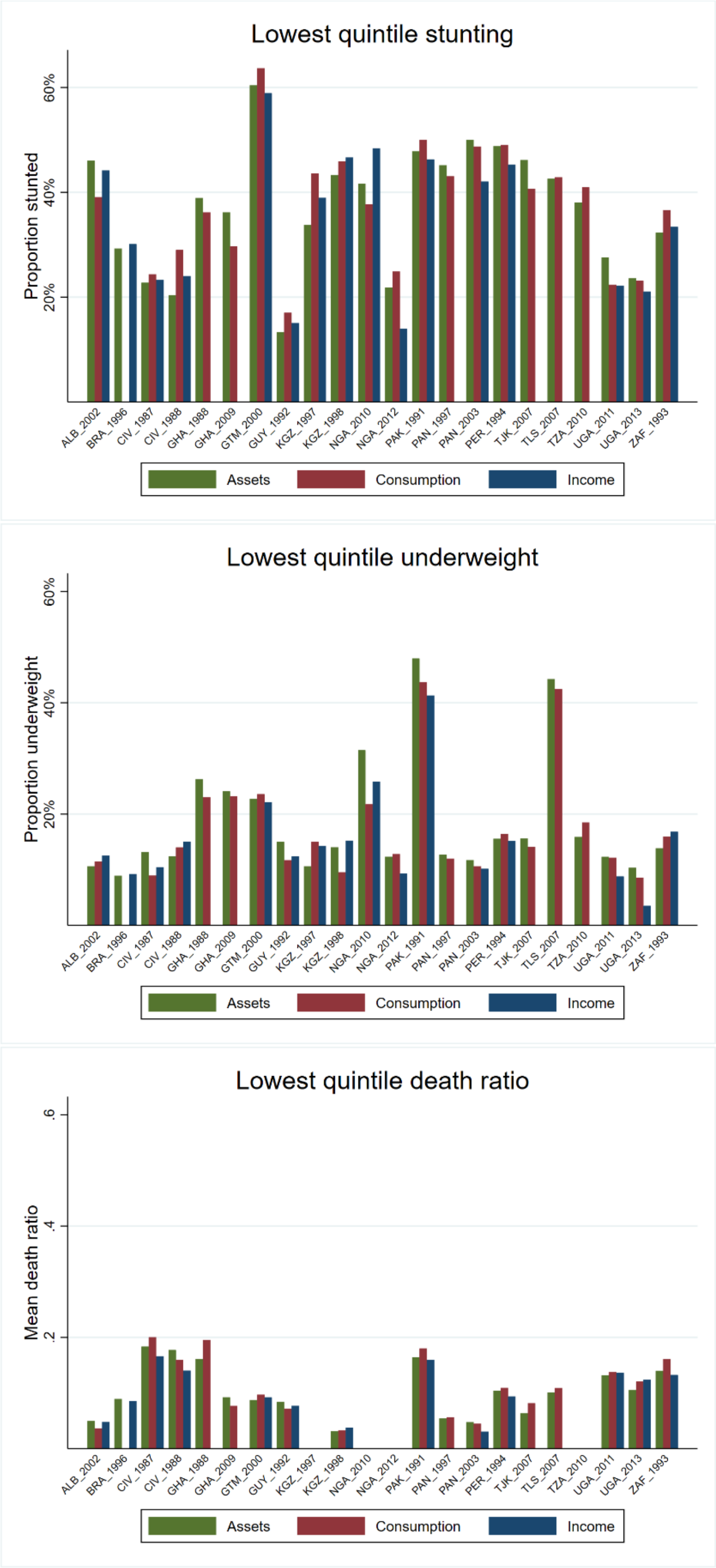

Table S3. Stunting concentration index values for each survey wave with income, consumption, and asset index comparison.

		Income					Consumption					Assets				
Country	Year	Obs.	CI	Std. error	UCI	LCI	Obs.	CI	Std. error	UCI	LCI	Obs.	CI	Std. error	UCI	LCI
Albania	2002	1338	-0.08	0.03	-0.02	-0.14	1338	-0.01	0.03	0.05	-0.07	1333	-0.03	0.03	0.03	-0.09
Brazil	1996	1647	-0.21	0.04	-0.14	-0.28						1762	-0.25	0.03	-0.18	-0.31
Cote d'Ivoire	1987	2215	-0.12	0.03	-0.06	-0.18	2233	-0.17	0.03	-0.11	-0.23	2206	-0.09	0.03	-0.03	-0.15
Cote d'Ivoire	1988	2105	-0.09	0.03	-0.02	-0.15	2121	-0.14	0.03	-0.08	-0.20	2117	-0.04	0.03	0.02	-0.10
Ghana	1988						2906	-0.07	0.02	-0.03	-0.12	2898	-0.19	0.02	-0.15	-0.24
Ghana	2009						2931	-0.07	0.02	-0.02	-0.11	2607	-0.18	0.02	-0.14	-0.23
Guatemala	2000	5743	-0.22	0.01	-0.19	-0.25	5743	-0.28	0.01	-0.25	-0.31	5743	-0.28	0.01	-0.26	-0.31
Guyana	1992	601	-0.04	0.07	0.09	-0.17	601	-0.11	0.07	0.02	-0.24	601	0.03	0.07	0.16	-0.10
Kyrgyzstan	1997	1162	-0.06	0.04	0.00	-0.13	1163	-0.14	0.03	-0.08	-0.21	1113	0.01	0.04	0.08	-0.06
Kyrgyzstan	1998	1689	-0.06	0.03	-0.01	-0.12	1727	-0.08	0.03	-0.02	-0.13	1643	-0.02	0.03	0.04	-0.07
Nigeria	2010	282	-0.27	0.07	-0.13	-0.40	2576	-0.05	0.02	0.00	-0.09	2404	-0.15	0.02	-0.10	-0.19
Nigeria	2012	205	0.07	0.10	0.27	-0.12	2737	-0.04	0.03	0.02	-0.09	2766	-0.06	0.03	-0.01	-0.11
Pakistan	1991	3911	-0.12	0.02	-0.08	-0.15	3916	-0.09	0.02	-0.05	-0.13	3903	-0.14	0.02	-0.11	-0.18
Panama	1997						2294	-0.52	0.03	-0.46	-0.57	2292	-0.61	0.03	-0.55	-0.66
Panama	2003	2922	-0.24	0.02	-0.20	-0.29	2922	-0.35	0.02	-0.30	-0.39	2922	-0.44	0.02	-0.40	-0.48
Peru	1994	2296	-0.28	0.02	-0.23	-0.32	2296	-0.34	0.02	-0.30	-0.39	2228	-0.38	0.02	-0.33	-0.43
Tajikistan	2007						2701	-0.02	0.02	0.02	-0.07	2694	-0.15	0.02	-0.10	-0.19
Timor Leste	2007						3937	0.06	0.02	0.09	0.02	3937	0.04	0.02	0.07	0.00
Tanzania	2010						2971	-0.16	0.02	-0.12	-0.20	2993	-0.18	0.02	-0.13	-0.22
Uganda	2011	2276	-0.09	0.03	-0.03	-0.15	2511	-0.11	0.03	-0.05	-0.16	2504	-0.15	0.03	-0.09	-0.21
Uganda	2013	187	-0.17	0.12	0.06	-0.40	2583	-0.13	0.03	-0.08	-0.19	2580	-0.13	0.03	-0.07	-0.18
South Africa	1993	4876	-0.21	0.02	-0.17	-0.24	4960	-0.22	0.02	-0.18	-0.25	4967	-0.20	0.02	-0.16	-0.24
Median			-0.12					-0.11					-0.15			
Average			-0.14					-0.14					-0.16			
Max			0.07					0.06					0.04			
Min			-0.28					-0.52					-0.61			

Table S4. Underweight concentration index values for each survey wave with income, consumption, and asset index comparison.

		Income					Consumption					Assets				
Country	Year	Obs.	CI	Std. error	UCI	LCI	Obs.	CI	Std. error	UCI	LCI	Obs.	CI	Std. error	UCI	LCI
Albania	2002	1338	-0.15	0.05	-0.05	-0.26	1338	-0.06	0.05	0.04	-0.17	1333	-0.04	0.05	0.07	-0.14
Brazil	1996	1647	-0.24	0.06	-0.12	-0.36						1762	-0.28	0.06	-0.16	-0.39
Cote d'Ivoire	1987	2215	-0.06	0.04	0.02	-0.14	2233	-0.05	0.04	0.02	-0.13	2206	-0.11	0.04	-0.03	-0.19
Cote d'Ivoire	1988	2105	-0.05	0.04	0.03	-0.13	2121	-0.07	0.04	0.01	-0.15	2117	-0.10	0.04	-0.02	-0.18
Ghana	1988						2906	-0.05	0.03	0.00	-0.10	2898	-0.18	0.03	-0.13	-0.23
Ghana	2009						2931	-0.07	0.03	-0.02	-0.12	2607	-0.11	0.03	-0.06	-0.16
Guatemala	2000	5743	-0.20	0.02	-0.16	-0.24	5743	-0.24	0.02	-0.20	-0.28	5743	-0.26	0.02	-0.22	-0.30
Guyana	1992	601	-0.04	0.07	0.10	-0.18	601	-0.14	0.07	-0.01	-0.28	601	-0.06	0.07	0.08	-0.20
Kyrgyzstan	1997	1162	-0.15	0.06	-0.04	-0.26	1163	-0.13	0.06	-0.03	-0.24	1113	0.06	0.06	0.18	-0.05
Kyrgyzstan	1998	1689	0.02	0.04	0.10	-0.06	1727	0.05	0.04	0.13	-0.03	1643	-0.04	0.04	0.04	-0.12
Nigeria	2010	282	-0.22	0.08	-0.06	-0.37	2576	0.03	0.03	0.08	-0.02	2404	-0.14	0.03	-0.09	-0.19
Nigeria	2012	205	0.01	0.14	0.29	-0.27	2737	-0.06	0.03	0.00	-0.13	2766	-0.07	0.03	0.00	-0.14
Pakistan	1991	3911	-0.11	0.02	-0.08	-0.15	3916	-0.11	0.02	-0.07	-0.14	3903	-0.14	0.02	-0.11	-0.18
Panama	1997						2294	-0.43	0.05	-0.33	-0.53	2292	-0.51	0.05	-0.41	-0.61
Panama	2003	2922	-0.27	0.05	-0.18	-0.36	2922	-0.32	0.05	-0.23	-0.40	2922	-0.44	0.04	-0.36	-0.53
Peru	1994	2296	-0.33	0.04	-0.25	-0.41	2296	-0.35	0.04	-0.27	-0.43	2228	-0.39	0.04	-0.31	-0.48
Tajikistan	2007						2701	0.03	0.03	0.09	-0.03	2694	-0.06	0.03	0.00	-0.13
Timor Leste	2007						3937	0.01	0.02	0.04	-0.03	3937	-0.01	0.02	0.03	-0.04
Tanzania	2010						2971	-0.12	0.03	-0.06	-0.18	2993	-0.14	0.03	-0.08	-0.20
Uganda	2011	2276	-0.15	0.05	-0.06	-0.24	2511	-0.23	0.04	-0.14	-0.31	2504	-0.20	0.04	-0.12	-0.29
Uganda	2013	187	0.04	0.20	0.42	-0.35	2583	-0.13	0.04	-0.04	-0.21	2580	-0.20	0.04	-0.12	-0.29
South Africa	1993	4876	-0.16	0.02	-0.11	-0.21	4960	-0.14	0.02	-0.09	-0.18	4967	-0.11	0.02	-0.06	-0.16
Median			-0.15					-0.11					-0.13			
Average			-0.13					-0.12					-0.16			
Max			0.04					0.05					0.06			
Min			-0.33					-0.43					-0.51			

Table S5. Child deaths concentration index values for each survey wave with income, consumption, and asset index comparison.

		Income					Consumption					Assets				
Country	Year	Obs.	CI	Std. error	UCI	LCI	Obs.	CI	Std. error	UCI	LCI	Obs.	CI	Std. error	UCI	LCI
Albania	2002	2662	-0.25	0.04	-0.17	-0.32	2662	-0.18	0.04	-0.10	-0.25	2655	-0.22	0.04	-0.15	-0.30
Brazil	1996	3020	-0.31	0.03	-0.24	-0.37						3241	-0.34	0.03	-0.28	-0.40
Cote d'Ivoire	1987	1002	-0.16	0.04	-0.09	-0.23	1015	-0.23	0.04	-0.16	-0.30	1002	-0.25	0.04	-0.18	-0.32
Cote d'Ivoire	1988	1061	-0.08	0.03	-0.01	-0.15	1067	-0.12	0.03	-0.05	-0.19	1065	-0.19	0.03	-0.13	-0.26
Ghana	1988						1813	-0.11	0.02	-0.06	-0.15	1810	-0.12	0.02	-0.07	-0.16
Ghana	2009						2835	-0.13	0.03	-0.07	-0.18	2526	-0.26	0.03	-0.20	-0.32
Guatemala	2000	5929	-0.14	0.02	-0.11	-0.18	5929	-0.18	0.02	-0.14	-0.22	5929	-0.17	0.02	-0.13	-0.21
Guyana	1992	936	-0.13	0.05	-0.03	-0.22	936	-0.07	0.05	0.02	-0.16	936	-0.04	0.05	0.05	-0.14
Kyrgyzstan	1998	2377	-0.09	0.04	0.00	-0.18	2433	0.00	0.04	0.08	-0.09	2326	-0.03	0.04	0.05	-0.12
Pakistan	1991	4557	-0.11	0.02	-0.08	-0.14	4564	-0.13	0.02	-0.10	-0.16	4533	-0.15	0.02	-0.12	-0.18
Panama	1997						3495	-0.35	0.04	-0.27	-0.42	3487	-0.37	0.04	-0.29	-0.44
Panama	2003	4522	-0.09	0.04	-0.02	-0.16	4522	-0.22	0.04	-0.15	-0.29	4521	-0.25	0.04	-0.17	-0.32
Peru	1994	2922	-0.22	0.03	-0.17	-0.28	2922	-0.25	0.03	-0.20	-0.31	2841	-0.27	0.03	-0.21	-0.32
Tajikistan	2007						4481	-0.08	0.03	-0.03	-0.13	4454	-0.04	0.03	0.01	-0.09
Timor Leste	2007						3258	-0.14	0.02	-0.10	-0.19	3258	-0.14	0.02	-0.09	-0.19
Uganda	2011	1602	-0.14	0.03	-0.09	-0.19	1748	-0.13	0.03	-0.08	-0.18	1753	-0.13	0.03	-0.08	-0.18
Uganda	2013	152	-0.07	0.09	0.11	-0.25	2129	-0.12	0.02	-0.07	-0.16	2125	-0.08	0.02	-0.03	-0.13
South Africa	1993	6828	-0.19	0.02	-0.15	-0.22	6940	-0.23	0.02	-0.19	-0.26	6964	-0.21	0.02	-0.17	-0.24
Median			-0.14					-0.13					-0.18			
Average			-0.15					-0.16					-0.18			
Max			-0.07					0.00					-0.03			
Min			-0.31					-0.35					-0.37			

Table S6. Stunting RII values for each survey wave with income, consumption, and hybrid income proxy comparison.

		Income					Consumption					Hybrid				
Country	Year	RII	Std. error	LCI	UCI	Obs.	RII	Std. error	LCI	UCI	Obs.	RII	Std. error	LCI	UCI	Obs.
Albania	2002	1.30	0.14	1.05	1.59	1,338	1.03	0.11	0.84	1.26	1,338	1.12	0.12	0.90	1.39	1,333
Brazil	1996	2.86	0.50	2.03	4.04	1,647						3.44	0.59	2.46	4.80	1,762
Cote d'Ivoire	1987	1.80	0.29	1.32	2.46	2,215	2.30	0.36	1.69	3.13	2,233	1.56	0.25	1.14	2.13	2,206
Cote d'Ivoire	1988	1.52	0.24	1.12	2.06	2,105	1.97	0.31	1.45	2.68	2,121	1.23	0.19	0.90	1.67	2,117
Ghana	1988						1.32	0.12	1.11	1.57	2,906	2.02	0.17	1.70	2.38	2,898
Ghana	2009						1.33	0.13	1.10	1.62	2,931	2.19	0.24	1.78	2.70	2,607
Guatemala	2000	1.86	0.08	1.71	2.03	5,743	2.20	0.10	2.02	2.40	5,743	2.15	0.09	1.97	2.34	5,743
Guyana	1992	1.24	0.43	0.63	2.46	601	1.77	0.62	0.90	3.51	601	0.86	0.30	0.44	1.69	601
Kyrgyzstan	1997	1.27	0.17	0.98	1.65	1,162	1.70	0.22	1.32	2.20	1,163	0.98	0.13	0.76	1.27	1,113
Kyrgyzstan	1998	1.25	0.13	1.02	1.52	1,689	1.31	0.13	1.07	1.59	1,727	1.06	0.11	0.87	1.29	1,643
Nigeria	2010	2.42	0.61	1.48	3.96	282	1.20	0.11	1.00	1.43	2,576	1.75	0.17	1.45	2.10	2,404
Nigeria	2012	1.08	0.31	0.62	1.89	2,794	1.19	0.15	0.93	1.52	2,737	1.32	0.17	1.03	1.68	2,766
Pakistan	1991	1.44	0.09	1.28	1.62	3,911	1.35	0.08	1.20	1.53	3,916	1.58	0.10	1.40	1.78	3,903
Panama	1997						16.45	2.75	11.85	22.84	2,294	27.78	4.72	19.92	38.75	2,292
Panama	2003	2.86	0.29	2.34	3.49	2,922	4.64	0.49	3.79	5.70	2,922	7.83	0.85	6.34	9.68	2,922
Peru	1994	2.93	0.30	2.41	3.58	2,296	3.79	0.38	3.12	4.62	2,296	4.30	0.44	3.52	5.24	2,228
Tajikistan	2007						1.08	0.09	0.92	1.28	2,701	1.72	0.15	1.46	2.03	2,694
Timor Leste	2007						0.83	0.05	0.74	0.94	3,937	0.88	0.05	0.78	0.99	3,937
Tanzania	2010						1.94	0.18	1.61	2.33	2,971	2.01	0.18	1.68	2.40	2,993
Uganda	2011	1.54	0.22	1.17	2.04	2,276	1.67	0.23	1.28	2.18	2,511	2.04	0.28	1.56	2.67	2,504
Uganda	2013	2.44	1.52	0.72	8.27	187	1.86	0.25	1.44	2.42	2,583	1.80	0.24	1.39	2.33	2,580
South Africa	1993	2.46	0.21	2.08	2.89	4,876	2.64	0.22	2.24	3.12	4,960	2.41	0.20	2.05	2.84	4,967
Median		1.67					1.70					1.77				
Average		1.89					2.55					3.27				
Max		2.93					16.45					27.78				
Min		1.08					0.83					0.86				

Table S7. Underweight RII values for each survey wave with income, consumption, and hybrid income proxy comparison.

		Income					Consumption					Hybrid				
Country	Year	RII	Std. error	LCI	UCI	Obs.	RII	Std. error	LCI	UCI	Obs.	RII	Std. error	LCI	UCI	Obs.
Albania	2002	2.29	0.64	1.32	3.97	1,338	1.41	0.39	0.82	2.44	1,338	1.21	0.34	0.70	2.09	1,333
Brazil	1996	4.06	1.47	2.00	8.27	1,647						5.21	1.86	2.59	10.47	1,762
Cote d'Ivoire	1987	1.37	0.30	0.90	2.09	2,215	1.33	0.29	0.88	2.03	2,233	1.80	0.40	1.17	2.78	2,206
Cote d'Ivoire	1988	1.29	0.28	0.84	1.99	2,105	1.46	0.32	0.95	2.24	2,121	1.73	0.38	1.13	2.65	2,117
Ghana	1988						1.27	0.15	1.00	1.61	2,906	2.20	0.26	1.74	2.78	2,898
Ghana	2009						1.41	0.17	1.11	1.78	2,931	1.68	0.22	1.30	2.16	2,607
Guatemala	2000	2.69	0.28	2.20	3.28	5,743	3.33	0.34	2.72	4.08	5,743	3.58	0.37	2.93	4.37	5,743
Guyana	1992	1.21	0.45	0.59	2.51	601	2.10	0.78	1.01	4.34	601	1.36	0.50	0.66	2.80	601
Kyrgyzstan	1997	2.30	0.70	1.26	4.19	1,162	2.10	0.64	1.15	3.82	1,163	0.71	0.22	0.39	1.30	1,113
Kyrgyzstan	1998	0.89	0.18	0.59	1.33	1,689	0.78	0.16	0.52	1.17	1,727	1.21	0.25	0.80	1.81	1,643
Nigeria	2010	2.52	0.90	1.25	5.07	282	0.88	0.09	0.71	1.09	2,576	1.83	0.21	1.46	2.29	2,404
Nigeria	2012	1.73	0.80	0.70	4.28	2,794	1.41	0.26	0.98	2.03	2,737	1.47	0.27	1.02	2.11	2,766
Pakistan	1991	1.50	0.11	1.31	1.72	3,911	1.49	0.11	1.29	1.71	3,916	1.71	0.12	1.48	1.97	3,903
Panama	1997						14.98	5.10	7.68	29.20	2,294	30.48	11.26	14.77	62.89	2,292
Panama	2003	4.91	1.34	2.87	8.38	2,922	6.53	1.82	3.78	11.29	2,922	17.01	5.24	9.31	31.09	2,922
Peru	1994	6.85	1.74	4.16	11.28	2,296	7.75	1.99	4.69	12.81	2,296	10.20	2.71	6.06	17.17	2,228
Tajikistan	2007						0.87	0.14	0.64	1.19	2,701	1.38	0.22	1.01	1.88	2,694
Timor Leste	2007						0.98	0.06	0.86	1.11	3,937	1.02	0.07	0.90	1.16	3,937
Tanzania	2010						1.87	0.31	1.36	2.58	2,971	2.05	0.33	1.49	2.80	2,993
Uganda	2011	2.28	0.59	1.37	3.78	2,276	3.71	0.93	2.27	6.08	2,511	3.11	0.77	1.91	5.07	2,504
Uganda	2013	0.82	0.91	0.09	7.27	187	2.05	0.50	1.27	3.30	2,583	3.15	0.78	1.94	5.13	2,580
South Africa	1993	2.27	0.29	1.76	2.92	4,876	2.04	0.26	1.59	2.63	4,960	1.75	0.22	1.36	2.25	4,967
Median		2.27					1.49					1.78				
Average		2.44					2.85					4.36				
Max		6.85					14.98					30.48				
Min		0.82					0.78					0.71				

Table S8. Child deaths RII values for each survey wave with income, consumption, and hybrid income proxy comparison.

		Income					Consumption					Hybrid				
Country	Year	RII	Std. error	LCI	UCI	Obs.	RII	Std. error	LCI	UCI	Obs.	RII	Std. error	LCI	UCI	Obs.
Albania	2002	4.47	1.80	2.03	9.84	2,662	2.87	1.12	1.33	6.17	2,662	3.75	1.50	1.72	8.19	2,655
Brazil	1996	6.47	2.08	3.44	12.16	3,020						8.17	2.55	4.43	15.06	3,241
Cote d'Ivoire	1987	2.37	0.71	1.32	4.27	1,002	3.52	1.08	1.93	6.42	1,015	3.95	1.22	2.16	7.23	1,002
Cote d'Ivoire	1988	1.54	0.44	0.88	2.70	1,061	1.87	0.54	1.06	3.30	1,067	2.82	0.82	1.59	5.00	1,065
Ghana	1988						1.74	0.35	1.17	2.58	1,813	1.82	0.37	1.23	2.70	1,810
Ghana	2009						2.04	0.53	1.23	3.38	2,835	4.51	1.33	2.53	8.03	2,526
Guatemala	2000	2.26	0.39	1.61	3.16	5,929	2.81	0.49	2.00	3.94	5,929	2.65	0.46	1.89	3.72	5,929
Guyana	1992	2.00	0.83	0.89	4.51	936	1.48	0.61	0.66	3.30	936	1.27	0.53	0.56	2.86	936
Kyrgyzstan	1998	1.69	0.67	0.78	3.68	2,377	1.03	0.39	0.49	2.17	2,433	1.23	0.48	0.57	2.63	2,326
Pakistan	1991	1.80	0.24	1.39	2.33	4,557	1.96	0.26	1.51	2.54	4,564	2.20	0.29	1.69	2.85	4,533
Panama	1997						9.00	3.87	3.88	20.90	3,495	10.36	4.51	4.41	24.34	3,487
Panama	2003	1.70	0.55	0.91	3.19	4,522	3.76	1.25	1.96	7.20	4,522	4.45	1.50	2.30	8.61	4,521
Peru	1994	3.58	0.90	2.18	5.87	2,922	4.30	1.10	2.60	7.11	2,922	4.76	1.24	2.86	7.93	2,841
Tajikistan	2007						1.60	0.33	1.07	2.40	4,481	1.25	0.26	0.84	1.88	4,454
Timor Leste	2007						2.24	0.49	1.45	3.45	3,258	2.18	0.48	1.42	3.36	3,258
Uganda	2011	2.09	0.53	1.28	3.42	1,602	1.96	0.48	1.22	3.16	1,748	1.95	0.47	1.22	3.13	1,753
Uganda	2013	1.50	1.45	0.23	10.00	152	1.93	0.47	1.20	3.12	2,129	1.57	0.37	0.99	2.51	2,125
South Africa	1993	2.76	0.36	2.14	3.56	6,828	3.51	0.46	2.71	4.54	6,940	3.13	0.41	2.42	4.04	6,964
Median		2.09					2.04					2.74				
Average		2.63					2.80					3.45				
Max		6.47					9.00					10.36				
Min		1.50					1.03					1.23				

Table S9. Stunting SII values for each survey wave with income, consumption, and hybrid income proxy comparison.

		Income					Consumption					Hybrid				
Country	Year	SII	Std. error	LCI	UCI	Obs.	SII	Std. error	LCI	UCI	Obs.	SII	Std. error	LCI	UCI	Obs.
Albania	2002	-0.12	0.05	-0.21	-0.03	1,338	-0.01	0.05	-0.11	0.08	1,338	-0.05	0.05	-0.14	0.04	1,333
Brazil	1996	-0.19	0.03	-0.26	-0.13	1,647						-0.22	0.03	-0.28	-0.16	1,762
Cote d'Ivoire	1987	-0.10	0.03	-0.16	-0.05	2,215	-0.15	0.03	-0.21	-0.10	2,233	-0.07	0.03	-0.13	-0.02	2,206
Cote d'Ivoire	1988	-0.08	0.03	-0.14	-0.02	2,105	-0.12	0.03	-0.18	-0.07	2,121	-0.04	0.03	-0.09	0.02	2,117
Ghana	1988						-0.10	0.03	-0.16	-0.04	2,906	-0.28	0.03	-0.34	-0.22	2,898
Ghana	2009						-0.09	0.03	-0.15	-0.03	2,931	-0.23	0.03	-0.29	-0.17	2,607
Guatemala	2000	-0.33	0.02	-0.37	-0.29	5,743	-0.42	0.02	-0.46	-0.38	5,743	-0.43	0.02	-0.47	-0.39	5,743
Guyana	1992	-0.03	0.05	-0.13	0.07	601	-0.09	0.05	-0.19	0.01	601	0.02	0.05	-0.08	0.12	601
Kyrgyzstan	1997	-0.09	0.05	-0.19	0.00	1,162	-0.21	0.05	-0.30	-0.11	1,163	0.01	0.05	-0.09	0.11	1,113
Kyrgyzstan	1998	-0.09	0.04	-0.17	-0.01	1,689	-0.11	0.04	-0.19	-0.03	1,727	-0.03	0.04	-0.11	0.06	1,643
Nigeria	2010	-0.41	0.10	-0.61	-0.22	282	-0.07	0.03	-0.13	0.00	2,576	-0.21	0.03	-0.27	-0.14	2,404
Nigeria	2012	-0.02	0.06	-0.13	0.10	2,794	-0.04	0.03	-0.09	0.02	2,737	-0.06	0.03	-0.12	-0.01	2,766
Pakistan	1991	-0.17	0.03	-0.23	-0.12	3,911	-0.13	0.03	-0.19	-0.08	3,916	-0.21	0.03	-0.27	-0.16	3,903
Panama	1997															
Panama	2003	-0.30	0.03	-0.36	-0.25	2,922	-0.41	0.03	-0.47	-0.36	2,922	-0.49	0.02	-0.54	-0.44	2,922
Peru	1994	-0.38	0.03	-0.44	-0.32	2,296	-0.47	0.03	-0.54	-0.41	2,296	-0.53	0.03	-0.59	-0.47	2,228
Tajikistan	2007						-0.03	0.03	-0.10	0.03	2,701	-0.21	0.03	-0.27	-0.15	2,694
Timor Leste	2007						0.08	0.03	0.03	0.14	3,937	0.06	0.03	0.00	0.11	3,937
Tanzania	2010						-0.21	0.03	-0.26	-0.15	2,971	-0.25	0.03	-0.30	-0.19	2,993
Uganda	2011	-0.10	0.03	-0.15	-0.04	2,276	-0.11	0.03	-0.17	-0.06	2,511	-0.15	0.03	-0.21	-0.10	2,504
Uganda	2013	-0.11	0.08	-0.27	0.05	187	-0.14	0.03	-0.20	-0.08	2,583	-0.14	0.03	-0.19	-0.08	2,580
South Africa	1993	-0.25	0.02	-0.30	-0.21	4,876	-0.26	0.02	-0.30	-0.22	4,960	-0.24	0.02	-0.28	-0.20	4,967
Median		-0.11					-0.12					-0.21				
Average		-0.17					-0.15					-0.18				
Max		-0.02					0.08					0.06				
Min		-0.41					-0.47					-0.53				

Table S10. Underweight SII values for each survey wave with income, consumption, and hybrid income proxy comparison.

		Income					Consumption					Hybrid				
Country	Year	SII	Std. error	LCI	UCI	Obs.	SII	Std. error	LCI	UCI	Obs.	SII	Std. error	LCI	UCI	Obs.
Albania	2002	-0.09	0.03	-0.15	-0.04	1,338	-0.04	0.03	-0.10	0.02	1,338	-0.02	0.03	-0.08	0.04	1,333
Brazil	1996	-0.08	0.02	-0.11	-0.04	1,647						-0.08	0.02	-0.11	-0.05	1,762
Cote d'Ivoire	1987	-0.04	0.02	-0.08	0.01	2,215	-0.03	0.02	-0.08	0.01	2,233	-0.06	0.02	-0.10	-0.01	2,206
Cote d'Ivoire	1988	-0.03	0.02	-0.07	0.02	2,105	-0.04	0.02	-0.08	0.01	2,121	-0.06	0.02	-0.10	-0.01	2,117
Ghana	1988						-0.06	0.03	-0.11	0.00	2,906	-0.21	0.03	-0.26	-0.15	2,898
Ghana	2009						-0.08	0.03	-0.13	-0.03	2,931	-0.12	0.03	-0.17	-0.06	2,607
Guatemala	2000	-0.18	0.02	-0.21	-0.15	5,743	-0.22	0.02	-0.25	-0.19	5,743	-0.25	0.02	-0.28	-0.22	5,743
Guyana	1992	-0.03	0.05	-0.12	0.07	601	-0.11	0.05	-0.21	-0.01	601	-0.04	0.05	-0.14	0.05	601
Kyrgyzstan	1997	-0.08	0.03	-0.14	-0.02	1,162	-0.07	0.03	-0.13	-0.01	1,163	0.03	0.03	-0.03	0.09	1,113
Kyrgyzstan	1998	0.02	0.03	-0.04	0.07	1,689	0.04	0.03	-0.02	0.10	1,727	-0.03	0.03	-0.09	0.03	1,643
Nigeria	2010	-0.26	0.09	-0.43	-0.09	282	0.04	0.03	-0.02	0.10	2,576	-0.17	0.03	-0.23	-0.11	2,404
Nigeria	2012	-0.06	0.04	-0.14	0.03	2,794	-0.04	0.02	-0.08	0.00	2,737	-0.05	0.02	-0.09	0.00	2,766
Pakistan	1991	-0.16	0.03	-0.22	-0.11	3,911	-0.15	0.03	-0.21	-0.10	3,916	-0.20	0.03	-0.25	-0.15	3,903
Panama	1997															
Panama	2003	-0.09	0.01	-0.12	-0.06	2,922	-0.10	0.01	-0.12	-0.07	2,922	-0.11	0.01	-0.13	-0.09	2,922
Peru	1994	-0.14	0.02	-0.17	-0.11	2,296	-0.15	0.02	-0.19	-0.12	2,296					
Tajikistan	2007						0.02	0.02	-0.03	0.07	2,701	-0.05	0.02	-0.10	0.00	2,694
Timor Leste	2007						0.01	0.03	-0.04	0.06	3,937	-0.01	0.03	-0.06	0.04	3,937
Tanzania	2010						-0.08	0.02	-0.12	-0.04	2,971	-0.11	0.02	-0.15	-0.06	2,993
Uganda	2011	-0.06	0.02	-0.10	-0.03	2,276	-0.09	0.02	-0.13	-0.06	2,511	-0.08	0.02	-0.12	-0.05	2,504
Uganda	2013	0.02	0.08	-0.13	0.17	187	-0.06	0.02	-0.09	-0.02	2,583	-0.09	0.02	-0.12	-0.05	2,580
South Africa	1993	-0.12	0.02	-0.16	-0.09	4,876	-0.10	0.02	-0.13	-0.07	4,960	-0.09	0.02	-0.13	-0.06	4,967
Median		-0.08					-0.06					-0.08				
Average		-0.09					-0.07					-0.09				
Max		0.02					0.04					0.03				
Min		-0.26					-0.22					-0.25				

Table S11. Child deaths SII values for each survey wave with income, consumption, and hybrid income proxy comparison.

		Income					Consumption					Hybrid				
Country	Year	SII	Std. error	LCI	UCI	Obs.	SII	Std. error	LCI	UCI	Obs.	SII	Std. error	LCI	UCI	Obs.
Albania	2002	-0.04	0.01	-0.06	-0.02	2,662	-0.03	0.01	-0.06	-0.01	2,662	-0.04	0.01	-0.06	-0.02	2,655
Brazil	1996	-0.07	0.01	-0.09	-0.05	3,020						-0.08	0.01	-0.10	-0.06	3,241
Cote d'Ivoire	1987	-0.11	0.04	-0.17	-0.04	1,002	-0.14	0.03	-0.21	-0.08	1,015	-0.17	0.03	-0.23	-0.10	1,002
Cote d'Ivoire	1988	-0.06	0.04	-0.13	0.01	1,061	-0.07	0.03	-0.14	-0.01	1,067	-0.12	0.03	-0.19	-0.06	1,065
Ghana	1988						-0.08	0.03	-0.13	-0.02	1,813	-0.09	0.03	-0.14	-0.03	1,810
Ghana	2009						-0.05	0.02	-0.08	-0.02	2,835	-0.08	0.01	-0.11	-0.05	2,526
Guatemala	2000	-0.05	0.01	-0.07	-0.03	5,929	-0.07	0.01	-0.09	-0.05	5,929	-0.06	0.01	-0.09	-0.04	5,929
Guyana	1992	-0.06	0.03	-0.11	0.00	936	-0.03	0.03	-0.09	0.03	936	-0.02	0.03	-0.07	0.04	936
Kyrgyzstan	1998	-0.02	0.01	-0.04	0.01	2,377	0.00	0.01	-0.03	0.02	2,433	-0.01	0.01	-0.03	0.02	2,326
Pakistan	1991	-0.08	0.02	-0.11	-0.04	4,557	-0.08	0.02	-0.12	-0.05	4,564	-0.11	0.02	-0.15	-0.08	4,533
Panama	1997						-0.04	0.01	-0.05	-0.02	3,495	-0.04	0.01	-0.05	-0.03	3,487
Panama	2003	-0.01	0.01	-0.03	0.00	4,522	-0.03	0.01	-0.04	-0.01	4,522	-0.03	0.01	-0.05	-0.02	4,521
Peru	1994	-0.08	0.01	-0.11	-0.05	2,922	-0.08	0.01	-0.11	-0.06	2,922	-0.09	0.01	-0.11	-0.06	2,841
Tajikistan	2007						-0.03	0.01	-0.05	0.00	4,481	-0.01	0.01	-0.04	0.01	4,454
Timor Leste	2007						-0.05	0.01	-0.08	-0.02	3,258	-0.05	0.02	-0.08	-0.02	3,258
Uganda	2011	-0.08	0.03	-0.13	-0.03	1,602	-0.07	0.03	-0.12	-0.02	1,748	-0.08	0.03	-0.13	-0.03	1,753
Uganda	2013	-0.03	0.07	-0.16	0.11	152	-0.06	0.02	-0.10	-0.02	2,129	-0.04	0.02	-0.09	0.00	2,125
South Africa	1993	-0.10	0.01	-0.13	-0.08	6,828	-0.11	0.01	-0.14	-0.09	6,940	-0.11	0.01	-0.13	-0.08	6,964
Median		-0.06					-0.06					-0.07				
Average		-0.06					-0.06					-0.07				
Max		-0.01					0.00					-0.01				
Min		-0.11					-0.14					-0.17				

Figure S2. Concentration indices (top), RIIs (middle), and SIIs (bottom) for all outcomes, country-years, and SES measures with 95% confidence intervals

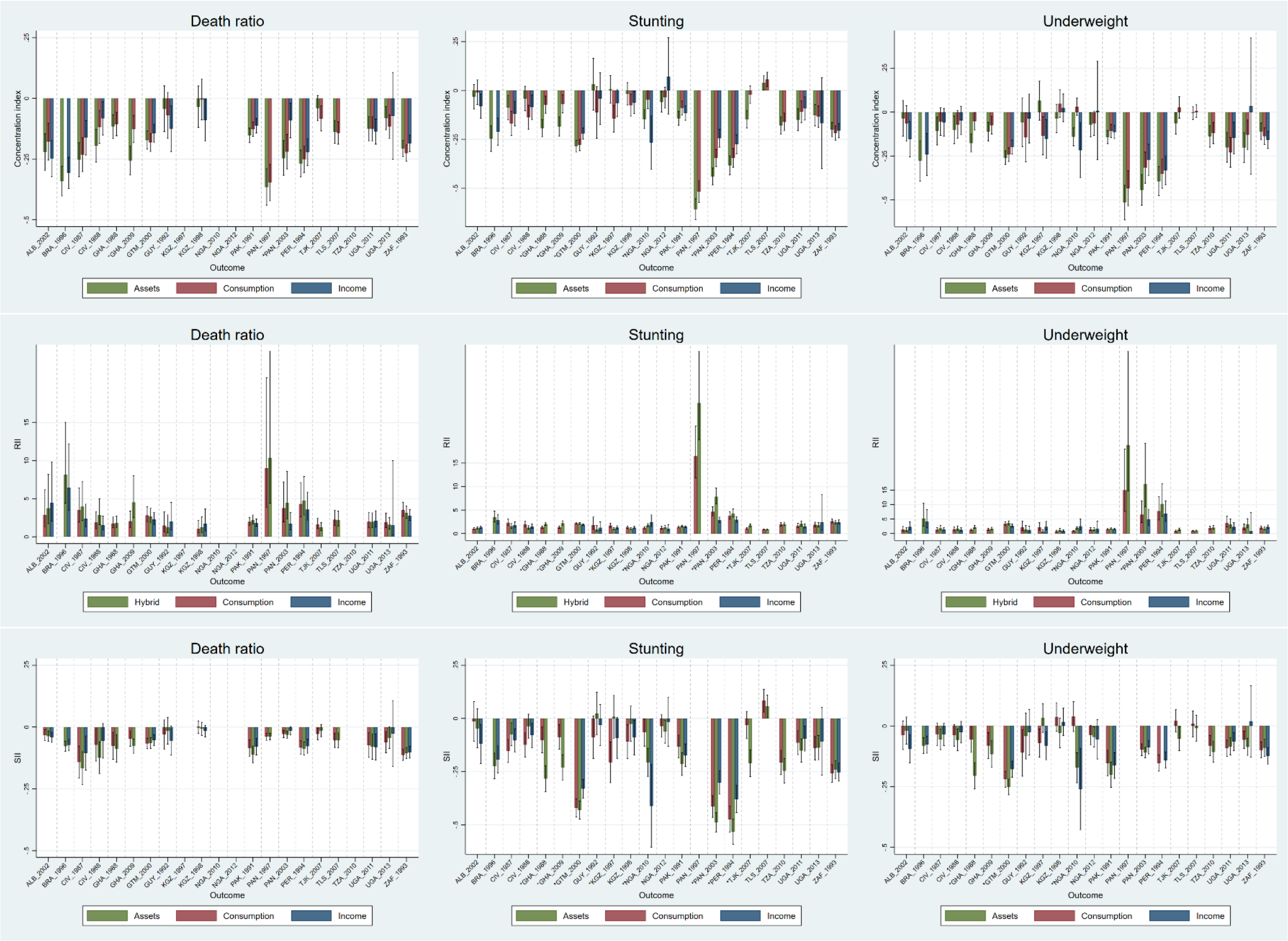

Figure S3. Forest plot comparisons of concentration indices using income, consumption, and assets for stunting (left), underweight (center), and child death ratios (right)

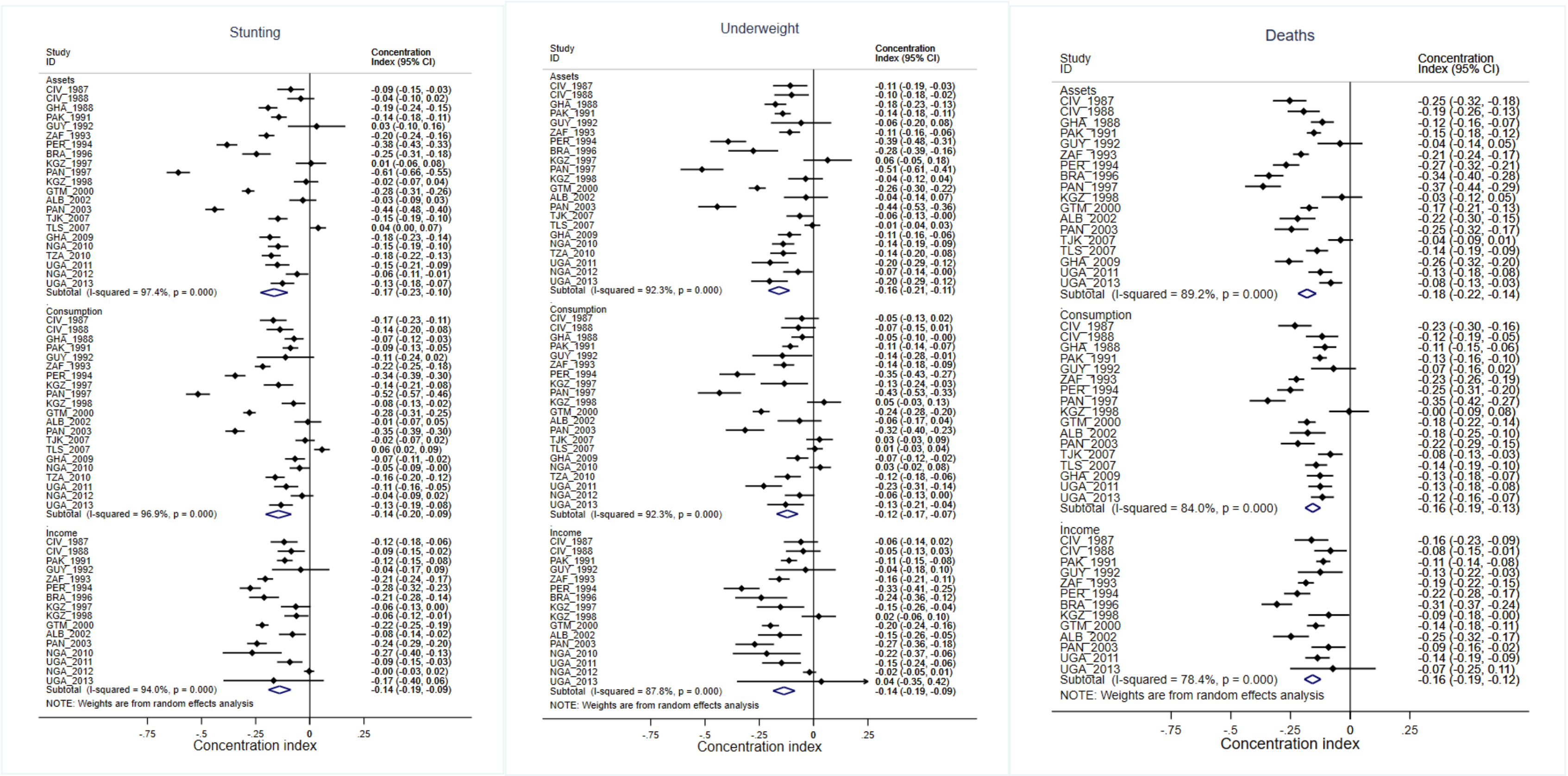

Figure S4. Forest plot comparisons of RII values using income, consumption, and income proxy hybrid for stunting (left), underweight (center), and child death ratios (right)

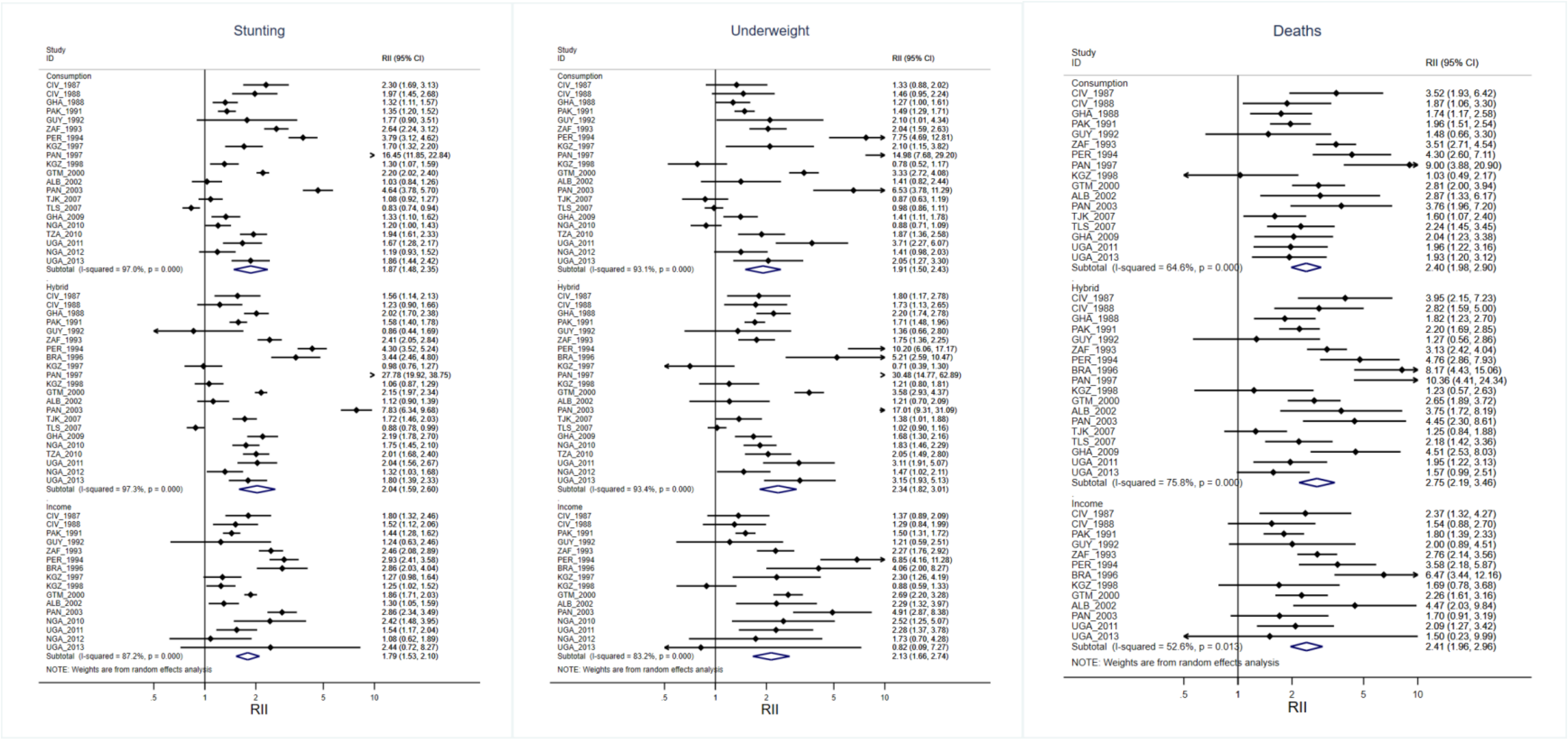

Figure S5. Forest plot comparisons of SII values using income, consumption, and income proxy hybrid for stunting (left), underweight (center), and child death ratios (right)

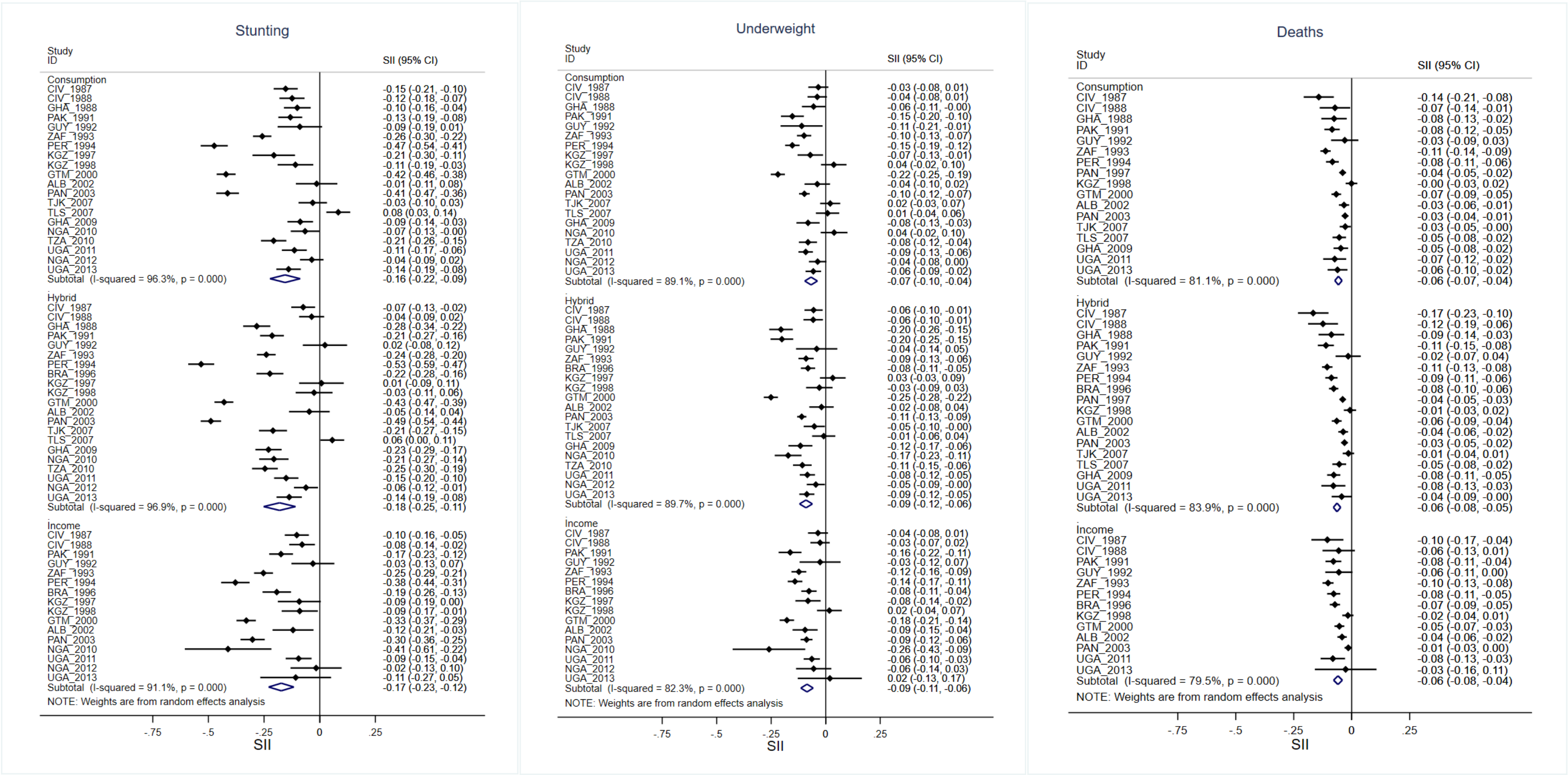

Supplement: Supplementary file 1 — Supplementary Information. [file 41598_2024_54170_MOESM1_ESM.pdf]
